# Impact of Sauropod Dinosaurs on Lagoonal Substrates in the Broome Sandstone (Lower Cretaceous), Western Australia

**DOI:** 10.1371/journal.pone.0036208

**Published:** 2012-05-25

**Authors:** Tony Thulborn

**Affiliations:** Kenmore, Queensland, Australia; Raymond M. Alf Museum of Paleontology, United States of America

## Abstract

Existing knowledge of the tracks left by sauropod dinosaurs (loosely ‘brontosaurs’) is essentially two-dimensional, derived mainly from footprints exposed on bedding planes, but examples in the Broome Sandstone (Early Cretaceous) of Western Australia provide a complementary three-dimensional picture showing the extent to which walking sauropods could deform the ground beneath their feet. The patterns of deformation created by sauropods traversing thinly-stratified lagoonal deposits of the Broome Sandstone are unprecedented in their extent and structural complexity. The stacks of transmitted reliefs (underprints or ghost prints) beneath individual footfalls are nested into a hierarchy of deeper and more inclusive basins and troughs which eventually attain the size of minor tectonic features. Ultimately the sauropod track-makers deformed the substrate to such an extent that they remodelled the topography of the landscape they inhabited. Such patterns of substrate deformation are revealed by investigating fragmentary and eroded footprints, not by the conventional search for pristine footprints on intact bedding planes. For that reason it is not known whether similar patterns of substrate deformation might occur at sauropod track-sites elsewhere in the world.

## Introduction

**Figure 1:**
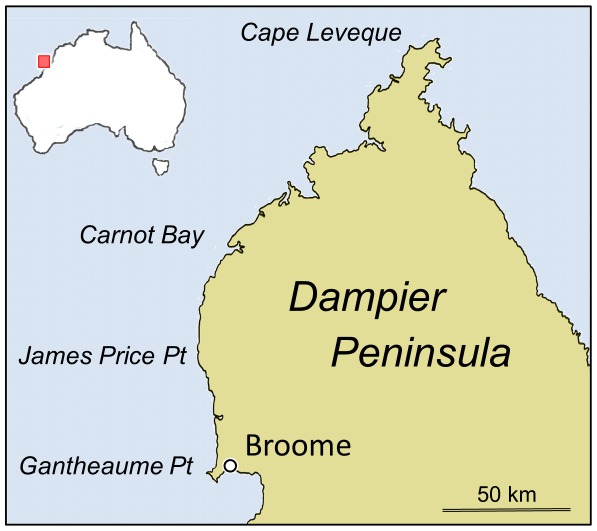
Map showing location of sites mentioned in text. The entire peninsula is composed of flat-lying beds of the Broome Sandstone (Early Cretaceous).

Before the 1990s there was very little evidence of dinosaurs in the western half of Australia. That vast geographic region, roughly equivalent in area to the western half of the continental USA, had produced only a few reports of some three-toed dinosaur tracks in sandstone beds at Gantheaume Point (Minyirr), near the town of Broome [1], [2], in the remote Kimberley region of Western Australia. Subsequently the sandstones at Gantheaume Point were designated the type section of the Broome Sandstone unit and were estimated to be of Early Cretaceous age (probably Valanginian, c. 130–135 My). The near-horizontal beds of the Broome Sandstone underlie the whole of the Dampier Peninsula, to the north of Broome (Figure 1), but there are few inland exposures and the unit is accessible mainly in a string of headlands and rocky foreshores along the peninsula’s western coast [2]–[7]. By the 1990s it was apparent that those coastal exposures of the Broome Sandstone contain a rich dinosaurian ichnofauna, including the tracks of sauropods, theropods, ornithopods and quadrupedal ornithischians provisionally identified as thyreophorans (armoured dinosaurs, perhaps stegosaurs) [8]–[11]. Ongoing research has revealed at least 16 distinct morphological types of dinosaur tracks in the Broome Sandstone, some referable to existing ichnotaxa and others certainly indicative of new ones. By world standards this is an outstandingly rich and diverse dinosaurian ichnofauna, and as sites elsewhere in Western Australia have yielded only a few fragments of dinosaur bone [12], [13], the Broome Sandstone remains the principal source of information about dinosaurs in this region of the globe.

**Figure 2:**
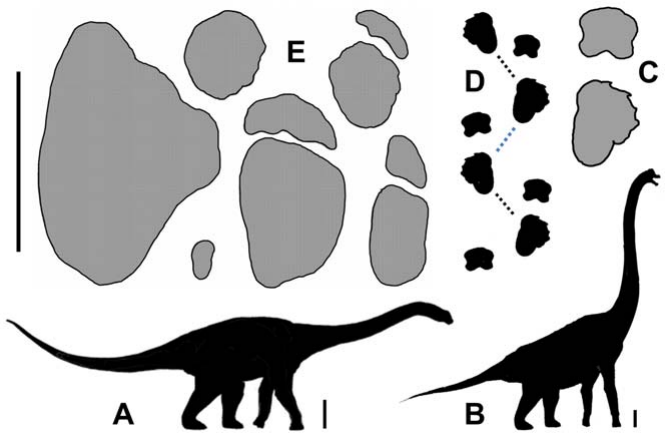
Cretaceous sauropod tracks and their potential rack-makers. A, silhouette of *Diamantinasaurus*, a titanosaur or related sauropod from the Winton Formation (Albian-Cenomanian) of Queensland (after Hocknull et al. [40]); scale bar indicates 1 metre. B, silhouette of *Brachiosaurus* (after Farlow [19]); undescribed skeletal fragments of a similar sauropod are also known to occur in the Rolling Downs Group of Queensland; scale bar indicates 1 metre. C, right manus-pes couple (at right) and D, part of a trackway (at left), of *Brontopodus birdi*, a distinctive form of sauropod track from the Trinity Group (Early Cretaceous, Comanchean) of Texas and Arkansas; after Farlow et al. [38]); long suspected to be the track of the contemporary brachiosaur *Pleurocoelus*, but more recently attributed [58] to *Paluxysaurus*, a relative of *Brachiosaurus*; length of the pes print ranges from 40–50 cm to more than 100 cm. E, a sample of sauropod tracks from the Broome Sandstone, Western Australia, to illustrate their diversity in size and shape; three isolated pes prints (at left) and three manus-pes couple (at right) are shown at uniform scale; scale bar (extreme left) is 1 metre.

The most abundant and conspicuous of the Broome Sandstone dinosaur tracks are those of sauropods (Figure 2), members of the clade Sauropoda, which included the biggest terrestrial animals of all time and are familiar to most people in the form of huge quadrupedal plant-eaters such as *Apatosaurus* (popularly known as *Brontosaurus*) and *Diplodocus* [14], [15]. The sauropod tracks in the Broome Sandstone are the first and only examples recorded in the Australasian region. Persistent reports of a sauropod footprint in the Walloon Coal Measures (Middle Jurassic, Bajocian) of Queensland (e.g.[16]–[19]) are erroneous. Some of them derive from a misreading of a catalogue of fossil reptiles in Queensland [20] whereas others refer to a ‘putative’ example which bears no resemblance to any known sauropod track and was originally attributed to a stegosaur [21]. That putative example clearly originated from an ornithischian dinosaur of some sort and is definitely not the work of a sauropod [22].

There are no such uncertainties about the sauropod tracks of the Broome Sandstone, which in some cases would qualify as textbook examples. The sauropod tracks have been reviewed in preliminary fashion elsewhere [9], and this present report is concerned not so much with the tracks themselves as with some of the remarkable sedimentary structures associated with them. Full-grown sauropods were big animals, sometimes estimated to have weighed 30–60 tons, or even more [14], [15], [19], and it is not surprising that they should have left all manner of disturbances in their wake, not just footprints. However, the extent to which a walking sauropod might deform the ground beneath its feet seems never to have been investigated very thoroughly. In fact, there is barely a mention of this subject in even the most comprehensive studies of the sauropod fossil record [19], [23]. By good fortune many sauropod tracks in the Broome Sandstone are preserved and exposed in a such a way that they show very clearly the patterns of disturbance and deformation that might be created by a walking sauropod. Some of those patterns appear to be unprecedented in their size and structural complexity, and they reveal that sauropods, like living elephants, were instrumental in remodelling the topography of their habitats.

### Palaeoenvironmental setting

**Figure 3:**
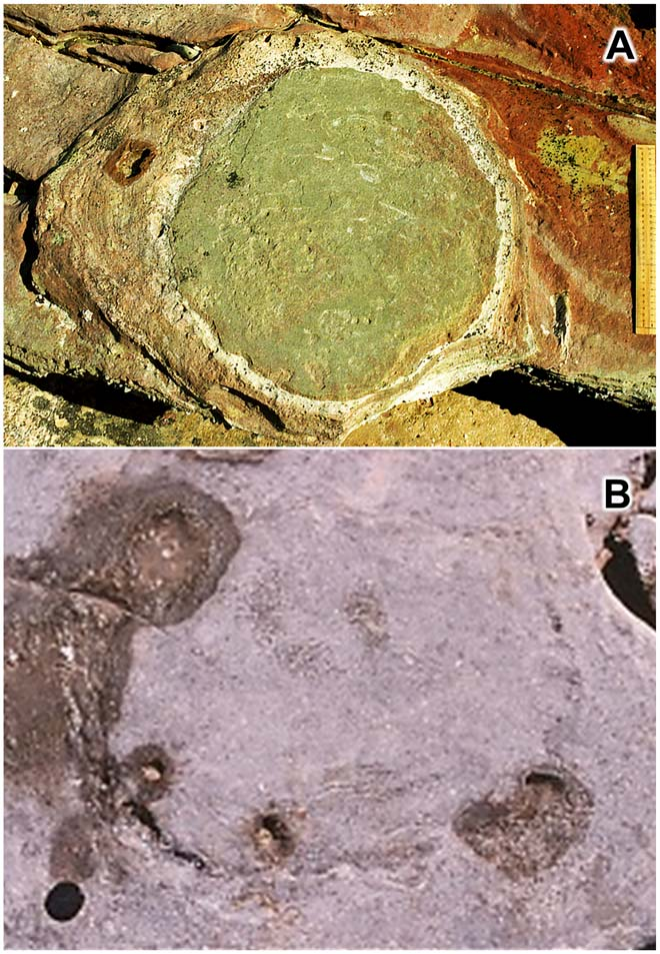
Variation in colour of Broome Sandstone and its sauropod dinosaur tracks. A, freshly-exposed and conspicuous example of a pes (hindfoot) print; the thinly layered sediments are characteristic of lagoonal substrates in the Broome Sandstone, though the vivid coloration is often subdued by weathering; scale is 1 ft (c. 31 cm) wooden ruler. B, pes print impressed in, and filled by, blue-grey siltstone; examples such as this are difficult to detect when sea-water has evaporated from the erosion pits along the interface between cast and mould; scale indicated by camera lens cap (diameter 6.7 cm) at lower left.

The sauropod tracks and sedimentary structures described below were observed in exposures of the Broome Sandstone at sites along the western coast of the Dampier Peninsula, which extends northwards from Broome for a distance of about 200 km (Figure 1). Only one of those scattered exposures, Gantheaume Point, near Broome, reveals a stratigraphic section thicker than 12–13 m, but borehole samples indicate that the Broome Sandstone attains a maximum thickness of at least 300 m [4], [6], [24], [25]. The base of the unit is seen only in borehole cores and its upper boundary is always an erosion surface. The Broome Sandstone is composed entirely of clastic sedimentary rocks, mainly fine-grained to coarse-grained sandstones, with subordinate siltstones and occasional conglomerates. There are also rare seams of pure white porcellanite, some thicker beds of greasy grey quartzite and irregular deposits of ironstone ranging in colour from red through purple and black. At several horizons there are dull brick-red palaeosols and carpets of silicified plant debris with stumps and roots of plants still in position of growth. The sandstones are often micaceous and are extremely varied in colour, ranging from vivid red, pink, orange, ochre and yellow through dull brown and grey (e.g. Figure 3). The cement is always siliceous or ferruginous, never calcareous. At some sites, such as Gantheaume Point, the sandstones exhibit large-scale cross-bedding, but at others, such as James Price Point (Walmadan), about 60 km north of Broome, they are more thinly and evenly bedded.

Aside from dinosaur tracks, the fossils reported to date from the Broome Sandstone include invertebrate trails, a few (arthropod?) burrows with meniscate fillings, arenaceous foraminiferans, microplankton, miospores and bivalves [6], [25], [26]. Plant remains are diverse and abundant, including araucarian conifers, cycads, ferns, bennettitalean seed-ferns and lycophytes, though there is no evidence of angiosperms or gingkos [27]–[29]. All the palaeontological evidence indicates that the sediments of the Broome Sandstone accumulated during the Early Cretaceous, with more precise estimates of age having ranged from Berriasian through Valanginian to Hauterivian (i.e. the ‘Neocomian’ of older literature). On balance the Broome Sandstone ichnofauna seems most likely to be of Valanginian age (c. 130–135 My) and, thus, roughly contemporaneous with the Wealden dinosaur faunas of southern England and Germany, but distinctly more ancient than the better-known dinosaur faunas of Early Cretaceous age (Aptian-Albian) in Queensland and Victoria [7], [30]. Dinosaur faunas of this particular age are poorly represented in the southern hemisphere and otherwise unknown in the Australasian region.

At the time the sediments of the Broome Sandstone were being deposited and traversed by dinosaurs, the Australian continental plate was still contiguous with Antarctica. The region identified today as India had just begun to detach from its southwestern margin, so that a narrow seaway, rather like the present-day Red Sea, intervened in the region of the Perth Basin. The sediments of the Broome Sandstone accumulated in a patchwork of environments along the northwestern margin of the continent [30], where the coastal plain was elaborated into a shifting patchwork of streams and channels, estuaries, deltas and swamps, with ephemeral lakes and patches of forest. To the seaward side lay extensive but very shallow lagoons which were occasionally flooded by run-off from the continental interior and periodically flushed by the tides. Patterns of banding in conifer wood indicate that the climate was markedly seasonal, and oxygen isotope studies [31] reveal that the region was somewhat warmer than the southeastern regions of Australia during the Early Cretaceous [28].

The sauropod dinosaur tracks are most conspicuous and most easily investigated in thinly-bedded sandstones and siltstones of lagoonal origin, where they are often associated with ripple-marks and invertebrate traces or, less commonly, with desiccation cracks. In places there is clear evidence of the very thin, wispy and sometimes lenticular bedding (flaser bedding) that is characteristic of sediments deposited under a tidal regime, but elsewhere the individual beds of sandstone and siltstone may reach a thickness of several centimetres. Often there is an alternating sequence of layers: fine-grained sandstones of dull bluish-grey colour alternate with darker siltstones whose brownish tinge presumably betrays a higher content of muddy terrigenous material. Those darker layers often succumb more rapidly to erosion, so that the more resistant sandstone layers are left projecting as paper-thin sheets.

At the time they were traversed by sauropods those lagoonal sediments were quite firm and cohesive. In a waterlogged or viscous substrate the walls of a footprint might be expected to slump inwards as the track-maker withdrew its foot, but very few of the sauropod tracks seen to date in the Broome Sandstone appear to have collapsed in that manner. Evidently the lagoonal substrates were sufficiently firm and cohesive that they could be moulded into deep indentations with free-standing vertical walls, rather like indentations in potter’s staple or modelling clay. The substrate was firm enough to support a ponderous sauropod, yet still sufficiently plastic to retain sharply-defined impressions of the animal’s feet. By virtue of its composition, comprising numerous thin sheets of silt and sand, it would register and retain patterns of sub-surface deformation that would not be apparent in more thickly-bedded and homogeneous substrates.

## Methods

Most tracks are exposed in the flat-lying beds of shore platforms, where the extreme tidal range, greater than 10 metres, permits only limited access - sometimes for intervals to be measured in minutes rather than hours. Even that limited access is unpredictable, as cyclones and storm surges transport vast quantities of sand and rubble along this dynamic and constantly-changing coast, burying some sites and exposing others at random. In practice these constraints mean that information must be gathered piecemeal and opportunistically. All the specimens illustrated here were studied *in situ* at intervals over the past 17 years. Most are far too large to be transported and placed in a reference collection, and removal of specimens is in any case prohibited by Australian National Heritage legislation (which applies to the entire western coast of the Dampier Peninsula, from Roebuck Bay to Cape Leveque).

**Figure 4:**
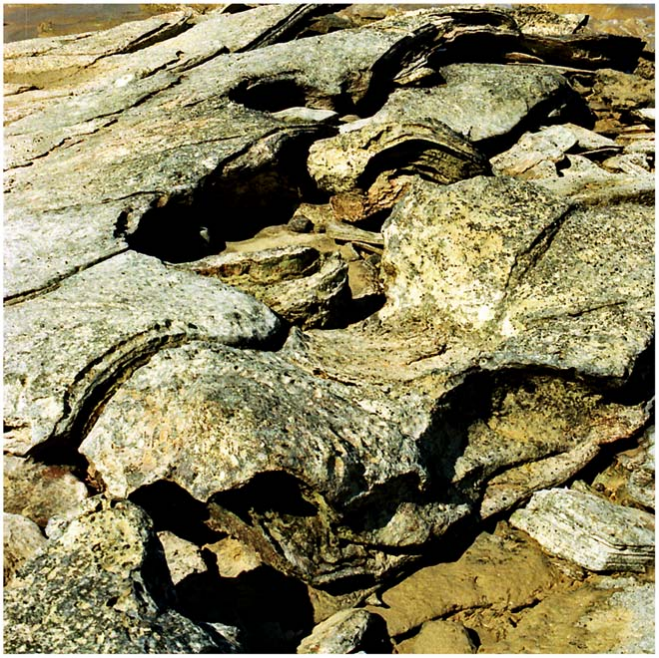
Part of a sauropod trackway in thinly-bedded lagoonal deposits of the Broome Sandstone. The deep footprints have controlled the development of fractures traversing the shore platform. Note the deep near-vertical walls of the prints.

Currently the ancient lagoonal deposits which contain the tracks are being exhumed by coastal erosion, practically undisturbed by tectonism and barely affected by the low regional dip (c. 2–3°). Those coastal sites which are sheltered from the direct impact of storm surges furnish the conventional, and essentially two-dimensional, view of sauropod tracks exposed on bedding planes [9], but the headlands and more exposed stretches of coast present a different picture, where the flat-lying beds of the shore platform are so shattered and deeply dissected that sauropod tracks are much less likely to survive intact. Here the deeply-impressed sauropod tracks are the agents of their own destruction, as they introduce points of structural weakness into the thinly-stratified rocks of the shore platform. Those deeply-sunken prints are analogous to a series of holes punched through a wad of paper - the predictable line of tearing through the paper, and the predictable line of fracturing and collapse when the shore platform is battered by waves (Figure 4). Conventional search for well-preserved or ‘museum-grade’ footprints exposed on bedding planes has found those much-eroded sites to be unrewarding [32]–[34], but that assessment needs to be qualified. From a different viewpoint such heavily-eroded sites are unusually informative: their numerous areas of natural breakage and erosion reveal a complementary three-dimensional view of the dinosaur tracks and provide some rare glimpses into the deeper regions of the substrates trodden by sauropods.

### Terminology

The term footprint (or print), referring to the natural mould or concave epirelief, will be restricted to that area of substrate impressed directly by the undersurface of a track-maker’s foot. This study uses the word footprint, rather than track, because it is accurate and unambiguous: it refers literally to the print of a foot and will, therefore, be readily understood in languages other than English. By contrast the widely-used term track is open to a variety of interpretations; it might refer to a single footprint or to a trackway (the series of footprints left by a single animal), or even to an accumulation of trackways left by numerous animals (as in colloquial expressions such as ‘off the beaten track’).

In all but the most superficial impressions the footprint will be limited by a boundary wall, which in more deeply-sunken prints will bear resemblance to the wall of a well or a vertical mine-shaft (Figure 4). For the purposes of this discussion the boundary wall, however high or low it may be, will not be regarded as part of the footprint *sensu stricto*. even though it is sometimes difficult to pinpoint an objective line of demarcation between the two. Similar conceptions of a footprint are evident in some other studies of dinosaur tracks (e.g. the ‘direct track’ of Gatesy [35] and the ‘true track’ of Lockley et al. [36], Milàn et al. [37]), though their authors did not always state explicitly that the boundary walls were to be excluded from the ambit of the footprint *sensu stricto*. Neither the natural cast (the footprint’s filling, a convex hyporelief protruding from the sole of the overlying bed) nor any surrounding or sub-surface feature will be regarded as part of the footprint *sensu stricto*. The utility of this seemingly pedantic definition will become apparent at a later point.

## Results

Well-preserved sauropod tracks have a very characteristic appearance and are unlikely to be mistaken for the tracks of any other dinosaurs [19], [22]. The finest examples of sauropod tracks are generally acknowledged to be some of those in the Glen Rose Formation (Trinity Group, Early Cretaceous) of Texas and Arkansas [19], [38], though it is now apparent that some specimens in the Broome Sandstone would certainly rival or surpass them in the quality of their preservation. However, the great majority of the world’s sauropod tracks are not so well-preserved and in many instances they are little more than featureless bowl-shaped depressions. Their size and their regular distribution in zig-zag trackway sequences may be the only indications that they are, indeed, the tracks of sauropod dinosaurs (assuming, of course, that they are found in sedimentary rocks of appropriate age).

**Figure 5:**
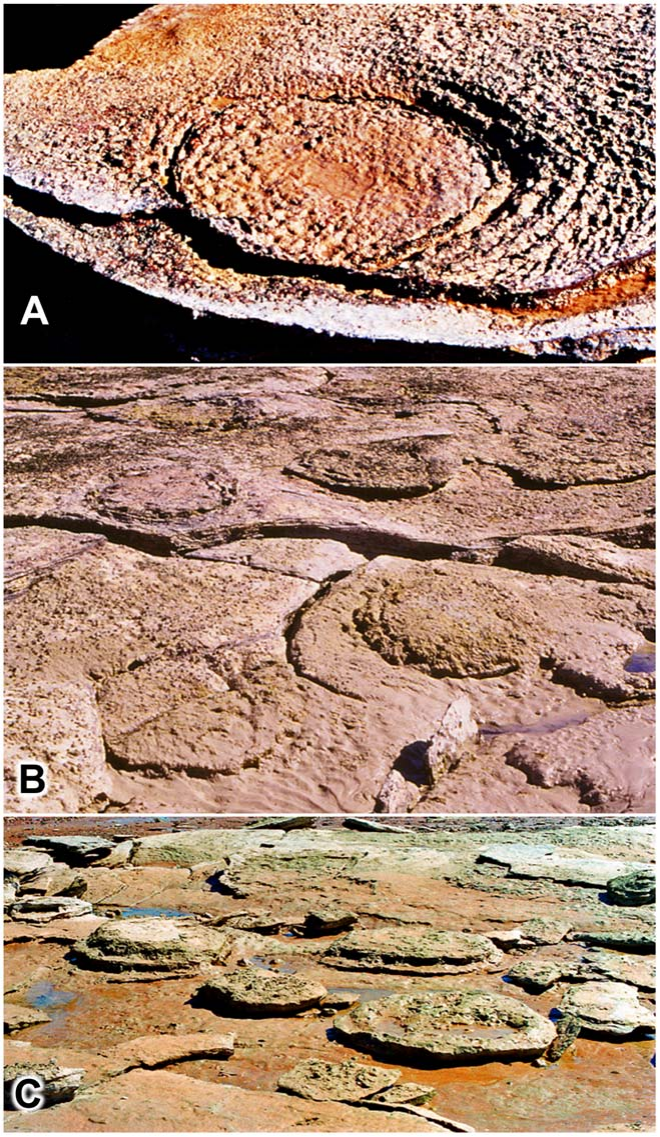
Variation in topographic expression of sauropod tracks in the Broome Sandstone. A, a shallow dish-like recess in exposed bedding plane (concave epirelief); the footprint’s filling is slightly more susceptible to erosion than the surrounding rock. B, with sediment filling being eroded at about same rate as the surrounding rock surface (see also Figure 3). C, footprints filled and capped by erosion-resistant filling persist as pedestals while less durable surrounding rock has been removed by erosion. All footprints shown are between 30 and 40 cm in length.

The sauropod tracks in the Broome Sandstone are frequently overlooked or misidentified, even by professional geologists and palaeontologists, and this is not just because of any shortcomings in their preservation. The difficulty arises for several reasons. First, and most importantly, the feet of sauropods do not resemble those of any other animals, living or extinct. Consequently the tracks of sauropods bear no likeness at all to the popular conception of a dinosaur’s footprint, which is commonly believed to be a ‘bird-like’ track with the marks of three large toes. On account of that pervasive misconception many sauropod tracks in the Broome Sandstone go unnoticed or are assumed to be erosional features or inorganic sedimentary structures. Second, the sauropod tracks sometimes escape notice because they are so diverse in their appearance: there is no single search-image. Some tracks are concave epireliefs (natural moulds; e.g. Figure 4), but many are partly or completely filled by the overlying sediment (e.g. Figure 3), and some are even represented by pedestals standing above the level of the surrounding rock (Figure 5). Finally there is a variety of circumstantial factors. As some footprints are accessible only at low tides, which may occur at any time of day or night, there can be no consistency in the angle or intensity of natural light. Even slight adjustments in the direction and intensity of lighting can have dramatic effects on the apparent size and shape of a dinosaur track (e.g. [39], figures 12A,B). A footprint that is sharply defined in late afternoon light may be harder to detect, or even invisible, in direct overhead lighting at mid-day; some tracks are visible when the rock is wet, but not when it is dry. For the same reasons there can be no uniformity in the various illustrations supplied here. In combination those several factors mean that any one sauropod track may look entirely different from another sauropod track.

Prints of the sauropod pes (hindfoot) are elephantine in form, roughly oval or subcircular in outline, but sometimes pear-shaped or subrectangular. Most examples in the Broome Sandstone are between 30 and 50 cm in length, though the smallest discovered to date are just over 20 cm whereas the largest are greater than 150 cm. When fully exposed, the floor of the pes print is rather flat, often with a distinctive downwards slope towards the more deeply-impressed medial margin. In well-preserved examples there may be a series of notches or pocket-like recesses formed by the large flat claws that wrapped around the anterior and antero-lateral rim of the track-maker’s foot. The number of claw impressions in the sauropod hindfoot prints is somewhat inconsistent; often there were three, though the footprints might not necessarily record a sharply-defined impression from each and every claw.

Although sauropods were habitual or obligate quadrupeds, their tracks do not necessarily include detectable prints of the manus (forefoot). In some instances the manus print was partly or completely overtrodden by the hindfoot, but in others its absence is simply a consequence of erosion, which destroys the relatively small manus prints much more rapidly than the deep pes prints. Even when prints of the manus are present, they tend to be less conspicuous than the pes prints and are easily overlooked. Usually the manus print lies directly ahead of the pes print and, sometimes, slightly off to the lateral side. It is generally smaller and less deeply-impressed than the pes print and is typically semi-circular or kidney-shaped in outline, with a convex leading edge. Often there are no clear indications of separate digits, which were bound together into a bundle and carried erect, so that the forelimb was supported virtually on the tips of its fingers. The sauropod manus typically has a single claw, on digit 1 (the pollex or ‘thumb’), but this is so rarely detected in prints of the manus that it is sometimes supposed to have been very small or entirely absent [19], [38]. Alternatively the absence of a claw-print may indicate that the claw was normally retracted and carried clear of the ground, like a cat’s claw [22]. Since the prints of the manus are usually so small and inconsistent in their occurrence, they will be mentioned only at those points where they seem to have any significant involvement in deforming the substrate.

Beyond those generalities, the sauropod tracks of the Broome Sandstone are so varied in their morphology that they might easily be classified in as many as four ichnotaxa plus a residue of anomalous forms. While it is easy to sort the tracks into such categories (e.g. Figure 2E), it seems practically impossible to ascertain the taxonomic identity of the sauropods that might have produced them [9], [38], A single sauropod might conceivably produce tracks belonging to two or more categories, depending on its circumstances and its style of locomotion, and it is equally possible that two unrelated sauropods might produce identical tracks, particularly if these are nothing more than featureless bowl-shaped depressions. In short, the exact identity of the sauropods responsible for the tracks remains something of a mystery. One sauropod bone has been discovered in Western Australia [12], but this is only an isolated caudal centrum of no special taxonomic significance. However, several species of sauropods are known to have existed in the eastern part of the continent during the Cretaceous, and in theory any of their relatives or ancestors might have been responsible for tracks in the Broome Sandstone. While the best-known of those sauropods may have been titanosaurs or, at least titanosaur relatives (cf. *Diamantinasaurus*, *Wintonotitan*, ?*Austrosaurus*; [40]), there is also evidence of some greater diversity, including undescribed skeletal fragments nearly identical to their counterparts in *Brachiosaurus*. Fortunately the exact identity of the Broome Sandstone sauropods is not particularly important in the present context.

### Adventitious features

Along with the footprints there are many incidental disturbances of the substrate. Their degree of development differs from place to place and was clearly governed by local circumstances, of which the most significant were the physical properties of the substrate and the size and behaviour of the track-maker. The adventitious features described below range from the strikingly obvious to the barely detectable: some are bigger and more conspicuous than the footprints themselves, and in places it is difficult to separate the footprints from the incidental disturbances that surround them.

The following account proceeds from the smaller and more superficial features to the more extensive and deeper-lying patterns of substrate deformation. It deals specifically with discrete sedimentary structures resulting from the impact of sauropod dinosaurs. Ill-defined areas of sediment trampled by sauropods and other dinosaurs occur quite commonly in the Broome Sandstone, but these will be mentioned only in connection with discrete sedimentary structures created by the activity of sauropods.

The development of adventitious features was governed by the responses of the substrate to the impact and penetration of the track-maker’s foot. The most significant responses were not necessarily those of the substrate as a whole, but those of the exposed surface and those of the individual layers of sediment composing the substrate. The degree to which the substrate resisted penetration by the track-maker’s foot would have been determined largely by factors such as density, cohesiveness and tensile strength, and in general terms each layer of sediment might respond to the impact of a sauropod’s foot in either of two ways: it might buckle, flex or contort but still retain its integrity (plastic deformation), or it might rupture, collapse or liquefy, thereby losing its integrity to some extent. In the first case the upper and lower boundaries of the sediment layer would remain intact, though the intervening sediment might be mobilized and redistributed. At any given point the overall thickness of the layer might be reduced by compaction (even to the point of zero thickness) or increased by influx from an adjoining area of compaction. In the second response at least one boundary is breached, so that sediment is transferred from one layer to another or extruded on to the exposed surface of the substrate. Although the following descriptions of sedimentary features tend to dwell on one or other of those responses, they are not mutually exclusive and are often found in conjunction.

### Peripheral displacements

The impact of the foot would inevitably displace the underlying and surrounding sediment, for otherwise there would be no detectable footprint. These peripheral displacements are expressed in a variety of minor topographic features, some more noticeable than others.

**Figure 6:**
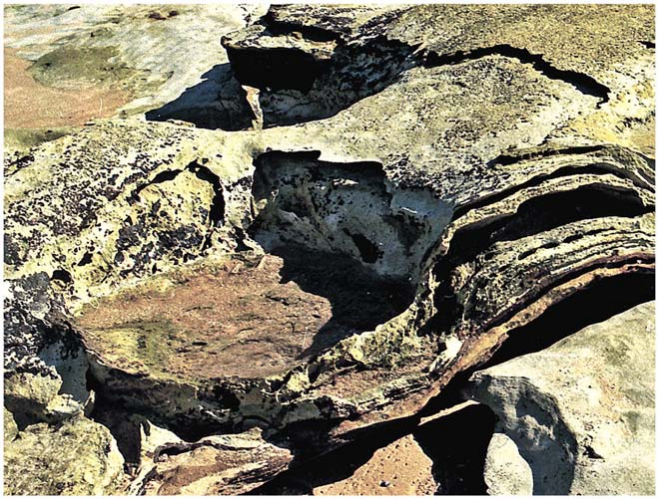
Interior of a deeply impressed sauropod pes print. Note the flat floor, representing the footprint *sensu stricto*, and the steep boundary wall (largely in shadow) which curves over at the top to merge into the undisturbed bedding plane. The smoothly curved transitions between the floor, the wall and the bedding plane make it extremely difficult to identify an objective limit for the footprint’s extent.

**Figure 7:**
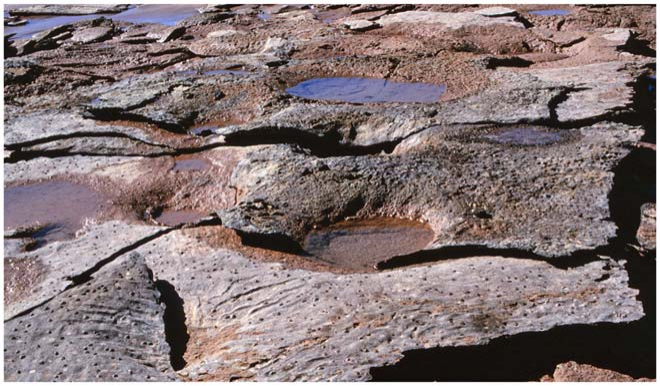
A scattering of sauropod tracks endows this bedding plane with an undulating appearance. The exact size of the individual prints is difficult to determine on account of their smoothly rounded margins. Note how the distribution of footprints has controlled the development of fractures through this paper-thin sheet of rock.

In places the lagoonal deposits trodden by the sauropods seem to have possessed a superficial skin of firm resilient sediment, perhaps dried by exposure to the air or reinforced by the growth of an algal film. Whatever its origin, this flexible skin clearly had sufficient tensile strength to offer some resistance to penetration by the track-maker’s foot. As the foot descended into the substrate, the surrounding area of the bedding plane would be dragged down into a conical depression analogous to the dimple that encloses the foot of an insect standing on water. The dimples created by a water-walking insect are temporary features, instantly eliminated by elastic recoil when the animal lifts its feet, but the equivalent depressions produced by the feet of sauropods were effectively permanent: they recoiled so slowly, or so incompletely, that they would be buried by the influx of more sediment and preserved along with the footprints they enclose. Consequently each sunken footprint lies at the floor of a well or vertical shaft with steeply-inclined walls that curve over at the top to merge into the exposed surface of the substrate (Figures 4,6). The transition between the footprint’s boundary wall (near-vertical) and the surface of the substrate (near-horizontal) is so smoothly rounded that it may be impossible to detect the precise extent to which the impact of the foot actually deformed the surface of the substrate. When sauropod pes prints are found in groups, as they often are, the smoothly rounded periphery of one print may merge into that of it neighbour, so that the entire bedding plane has an undulating appearance (e.g. Figure 7).

**Figure 8:**
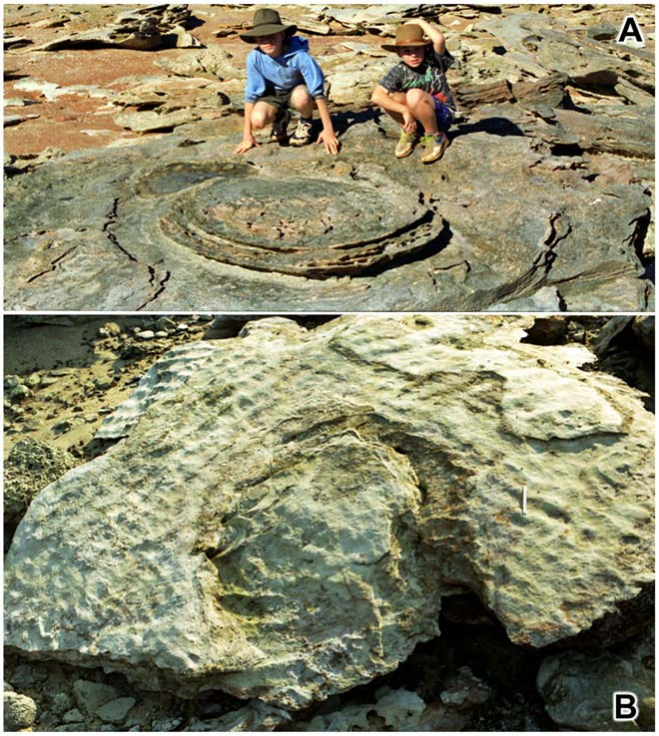
Lateral and superficial disturbances of substrate caused by impact of sauropod feet. A, shallow dish-like print surrounded by extensive ripple-like disturbances. The actual footprint (impressed directly by underside of the track-maker’s foot) has probably been lost to erosion, but nevertheless these sub-surface features convey a good idea of the extent to which impact of a sauropod’s foot could disturb the surrounding substrate. Note the very faint ripple-like disturbance at extreme left. B, undersurface of rock slab that formerly overlay and filled a sauropod pes print. Large oval feature at centre is that footprint’s rock filling (natural cast). The gutter surrounding it indicates that the original footprint was encircled by a raised rim of displaced sediment. Evidence of a second but much smaller print (the manus?) is at upper right, partly concealed by adherent rock. As the surface of this overturned slab is convex, it must have overlain a substrate that was concave - as explained diagrammatically in Figure 9. The regular dimpled texture results from two intersecting sets of ripple-marks. 10 cm scale to right of pes print.

The area of bedding plane disturbed by the impact of a single sauropod foot was sometimes more extensive than one might imagine, though (as yet) this can only be demonstrated indirectly and by fragmentary evidence, not by means of perfectly-preserved footprints on a pristine bedding plane. The first example (Figure 8A) is eroded to such an extent that the actual footprint (*sensu stricto*) has been destroyed and only some underlying parts of the substrate remain. Even so, it seems that relatively few and thin layers of rock have been stripped away, for the indentation in the uppermost layer still shows a sharp discontinuity between its floor and its wall. One would expect such a sharply-defined feature of relief to be detectable at or near the surface trodden by the track-maker but not at greater depths in the substrate. Overall, this specimen affords only a rough idea of the original footprint, but in the present context it is the surrounding rock which holds greater interest. This shows quite clearly that the impact of even a modest sauropod hindfoot could disturb a surprisingly large area of the surrounding substrate. In some specimens the ripple-like disturbances are detectable more than a metre away from the footprint. The lateral extent of those disturbances has remained unchanged since the Early Cretaceous, whereas their vertical extent has surely been reduced to some (unknown) extent by subsequent compaction of the substrate.

That particular pattern of disturbance, with low amplitude but great lateral extent, might not be entirely the result of substrate compaction and subsequent erosion: it might also betray the existence of a major discontinuity in the substrate. It is conceivable that the track-maker’s foot penetrated a relatively thin superficial zone of plastic sediment which was underlain by much firmer and more resistant material. While the foot itself produced only a shallow dish-like impression, its impact would have generated shock-waves spreading through the substrate in every direction. The horizontal component of force would spread through the superficial layer of sediment like a wave or ripple emerging concentrically from the planted foot, while the other components of force, directed obliquely and downwards, might have been reflected from the interface between the superficial (plastic) and underlying (firmer) sediments to reinforce the effects of the horizontal component.

**Figure 9:**
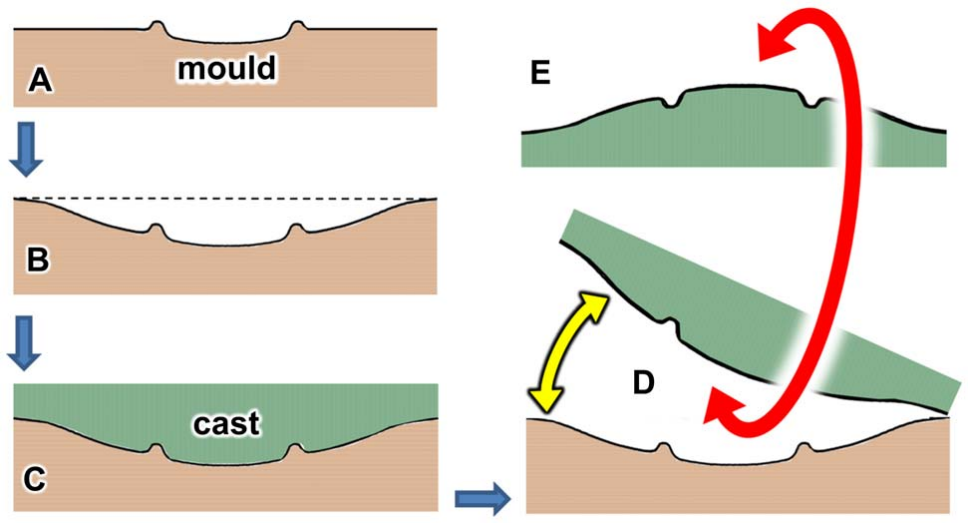
Series of diagrams explaining origin of the specimen shown in Figure 8B. A, sauropod footprint impressed into substrate; the footprint, a natural mould (concave epirelief) is bordered by a raised rim of displaced sediment. B, the footprint mould lies at the centre of a larger depression, apparently a zone of subsidence or down-warping created by the impact of the track-maker’s foot. C, the area is buried by an influx of sediment which fills the footprint mould to form the natural cast. D, much later, after lithification, the two layers of rock are separated by natural breakage and erosion. E, the upper layer is overturned by waves to expose its convex lower surface with the footprint cast surrounded by a gutter. Smaller features in Figure 8B (manus print and ripple-marks) are omitted for the sake of clarity.

The second example (Figure 8B) has been illustrated previously but was described incorrectly as a ‘sauropod underprint with a more weathering resistant core’ ([33], figure 8). It is, in fact, the sole of a sandstone slab which has been overturned by wave-action. The conspicuous elliptical feature at the centre of the slab is the natural rock filling of a sauropod pes print (i.e. the natural cast, or convex hyporelief), which was clearly encircled by a raised rim of displaced sediment - represented on the sole by a complementary gutter. Off to one side a shallow and much smaller marking, still obscured by some adherent rock, probably represents the associated print of the manus. This, too, was encircled by a raised rim of displaced sediment, represented on this complementary surface by a faint circular groove. In the present context, however, the most important feature of this specimen is the least obvious: its exposed surface is convex. This means, of course, that the complementary surface trodden by the track-maker must have been concave. In other words, the impact of the sauropod’s foot generated not only a footprint encircled by a rim of displaced sediment, but also a much more extensive depression (as explained in Figure 9). This last feature seems never to have been described previously, though there are indications of it elsewhere in the Broome Sandstone. Unfortunately it is not possible to determine the exact size and shape of this sunken zone around the footprint: it has no definite perimeter in the example shown here, and its extent is likely to remain unknown until a perfectly-preserved specimen can be discovered on a large area of intact bedding plane and examined under ideal conditions of low-angle lighting. The depressed area seems to decrease in depth as one traces it away from the footprint, and at some point it would, presumably, fade out entirely and merge insensibly into the undisturbed bedding plane. Such gradual fade-out might imply that the whole saucer-like depression is the result of down-warping, roughly analogous to the dimple created by the foot of an insect standing on water. Alternatively the discovery of a sharply-defined perimeter or marginal discontinuity might imply a different explanation, namely subsidence or collapse of the substrate. There is, in fact, a very faint suggestion of such a concentric step-like feature (towards lower left of slab shown in Figure 8B). Regardless of lingering uncertainty about its mode of origin, that large depression around the footprint clearly hints that some more extensive disturbance of the substrate is to be found sub-surface, beneath the exposed bedding plane.

**Figure 10:**
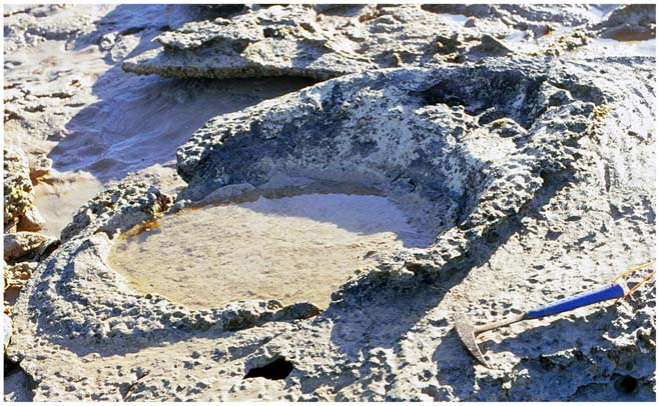
Manus-pes couple impressed into ripple-marked surface and surrounded by a raised rim of displaced sediment. Slightly obscured by modern wind-blown beach sand. The small shelf to right is a remnant of the shallow manus print, which was partly overtrodden and obliterated by the much bigger and deeper pes print. Scale indicated by geological hammer at lower right.

In traversing a thin superficial layer of plastic sediment underlain by firmer sediment, a sauropod would produce shallow dish-like print of the pes. Sediment displaced by the foot’s impact often welled up around the rim of the planted foot (Figure 10), though there may be little else in the way of visible disturbance. The depth to which a foot might sink into the substrate was governed partly by its shape [41], which also exerted some control on the transfer of displaced sediment into the elevated rim surrounding the footprint. Sediment squeezed outwards by the impact of the foot would accumulate in a zone bordering the footprint wall, and the length of that peripheral zone must vary according to the shape of the track-maker’s foot. For instance, a bird-like footprint with three salient toes has a much longer perimeter than does a sub-circular footprint of equal area. And if displaced sediment must be distributed along a shorter perimeter, it would naturally tend to produce a more pronounced marginal ridge. Thus, by virtue of their compact subcircular shape, the hindfeet of sauropods may have been predisposed to create prominent marginal ridges whenever they were impressed into suitable substrates.

Closer inspection reveals that there are two types of raised rim, probably reflecting two different conditions of the substrate. First, the substrate might have comprised a thin zone of plastic sediment overlying a deeper zone that was comparatively firm and unyielding. In effect the superficial layers of plastic sediment would have been trapped on a firm floor, where they might be trampled and churned by the feet of sauropods. The individual layers of sediment would be mashed into a slurry and squeezed out around the margins of the foot to form a raised rim defining the outline of a very flat and shallow footprint. That outcome occurred quite commonly when two or more animals happened to tread on a single spot.

**Figure 11:**
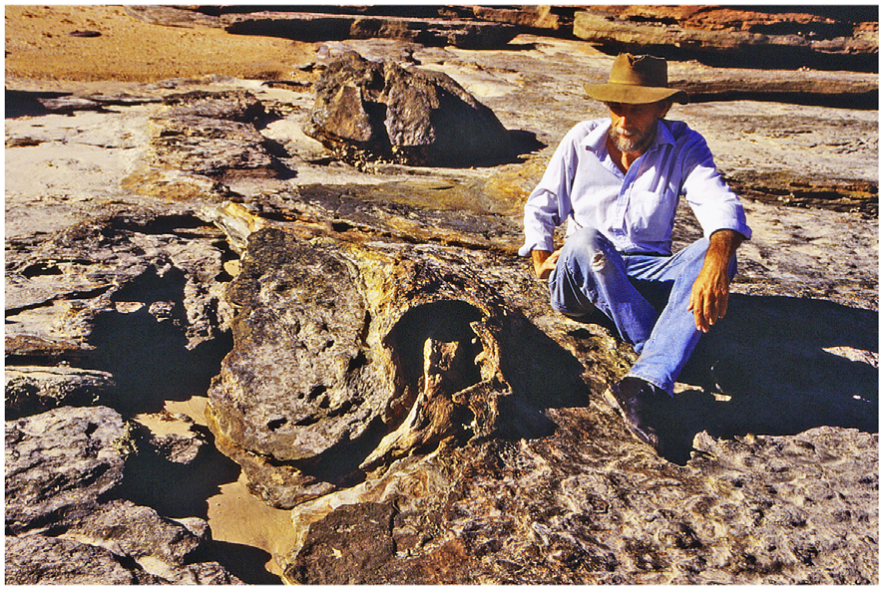
Thinly-layered lagoonal sediments pushed up into a rounded fold by a deeply-impressed sauropod footprint.

**Figure 12:**
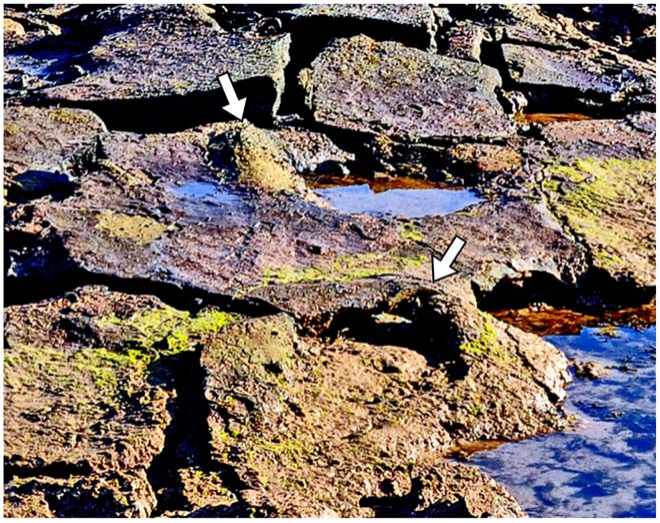
Sauropod pes prints each with raised rim of displaced sediment (arrowed) at its leading edge. Example in foreground is viewed from behind; example in mid-background shows the raised rim at front of pes (larger puddle) tilted forwards to overhang rear part of manus print (smaller puddle).

In a greater thickness of plastic sediments the foot would, of course, sink more deeply (even to a depth of more than 40 cm; e.g. Figure 11), and in these circumstances a second pattern was likely to emerge. Here, too, the footprint may be encircled by a raised rim, though in this case the individual layers of sediment retained their integrity and were not mixed into a slurry. As those layers were being squeezed and flattened beneath the planted foot. they would simultaneously inflate to form a distended rim around its margin. In some instances the wall of sediment pushed up by the leading edge of the pes was so pronounced that it toppled forwards to overhang the rear part of the manus print, thereby exaggerating its kidney-shaped outline (e.g. Figure 12).

### Subsidence

In places the impact of a sauropod’s foot was sufficient to cause localized subsidence of the substrate. The superficial layer(s) of sediment beneath and around the planted foot would subside, creating a roughly circular crater in the surface traversed by the dinosaur and a roughly corresponding protrusion from the sole(s) of the affected layer(s). Sometimes a central area of deep subsidence is encircled by a terrace that subsided to a lesser extent.

**Figure 13:**
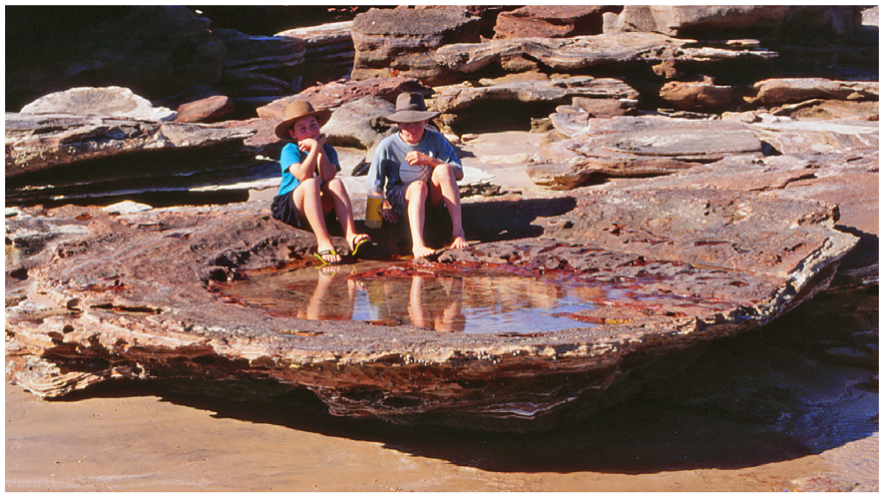
Crater-like area of subsidence produced by impact of sauropod feet. This example is situated high up on the beach, where extensive subaerial erosion has destroyed traces of sauropod track(s) in the interior - except possibly for some remnants (rounded notches) at extreme left. The flat interior and the steep boundary wall are characteristic features.

**Figure 14:**
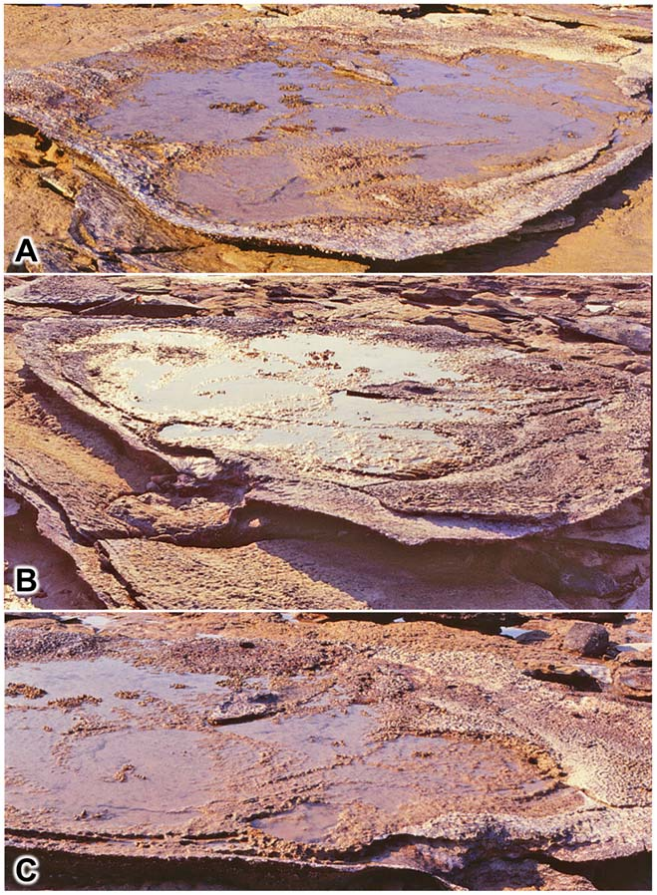
Basin of deformed substrate formed by sauropod trampling. A, the flat interior of the basin with shallow sauropod tracks, each outlined by a rim of displaced sediment. B, another view of the same example, showing curvature of underlying beds. C, closer view of one end, showing the steep boundary wall, overlapping pes prints and one clearly-defined manus print (centre foreground).

These areas of subsidence, or gigantic load casts, have a patchy distribution in the Broome Sandstone, and their development was presumably dependent on rather specific conditions of the substrate. Load casting would have required the density of the substrate’s superficial layer(s) to be in equilibrium with, or even slightly greater than, the density of the underlying layer(s). In those conditions a localized increase in loading, in the form of a sauropod’s planted foot, would be sufficient to trigger the process of load casting, where the mobilized superficial layer(s) of sediment would begin to spill down and intrude into the deeper-lying layer(s). The resulting sedimentary structures have a very distinctive appearance: they are large pan-shaped depressions of roughly circular or elliptical outline, bounded by a continuous wall which is fairly steep but no more than 20–30 cm in height. The floor of the depression is almost perfectly flat, and in freshly-exposed examples it may bear very shallow but clearly outlined impressions of sauropod footprints (though these tend to succumb rather rapidly to erosion). Small examples, about the size of a child’s paddling pool (c. 1–2 m, e.g. Figure 13), might conceivably be the work of a single sauropod, but larger basin-like features are definitely composite structures produced by two or more animals (e.g. Figure 14).

### Transmitted reliefs

**Figure 15:**
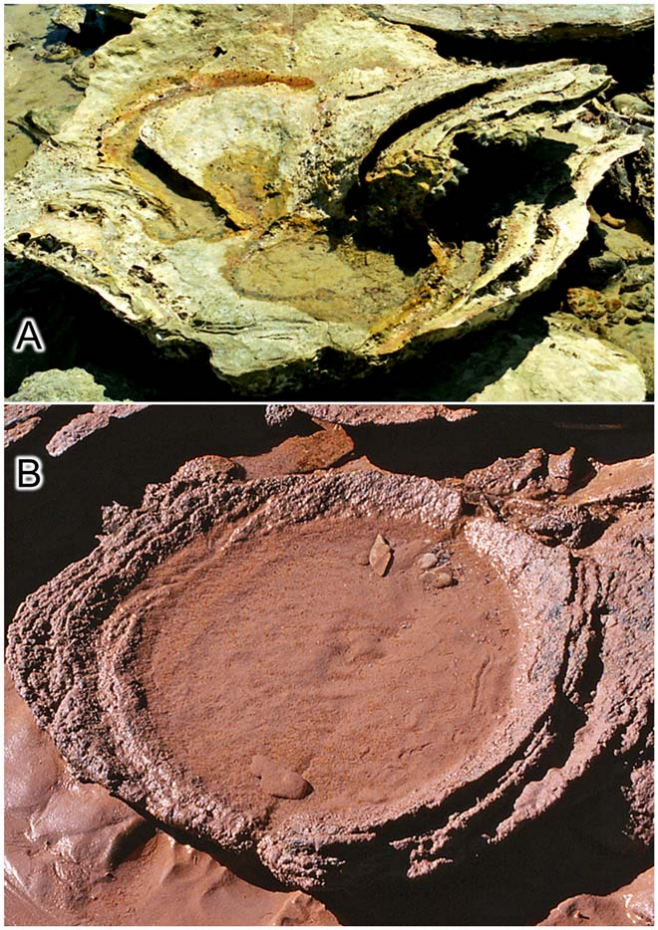
Sauropod footprints in thinly-layered sediments responsible for ‘onion-ring’ effect in broken or weathered specimens. A, example broken obliquely, showing extent of laminations enclosing the entire footprint. B, example broken horizontally, revealing typical ‘onion-ring’ pattern of laminations surrounding the footprint.

**Figure 16:**
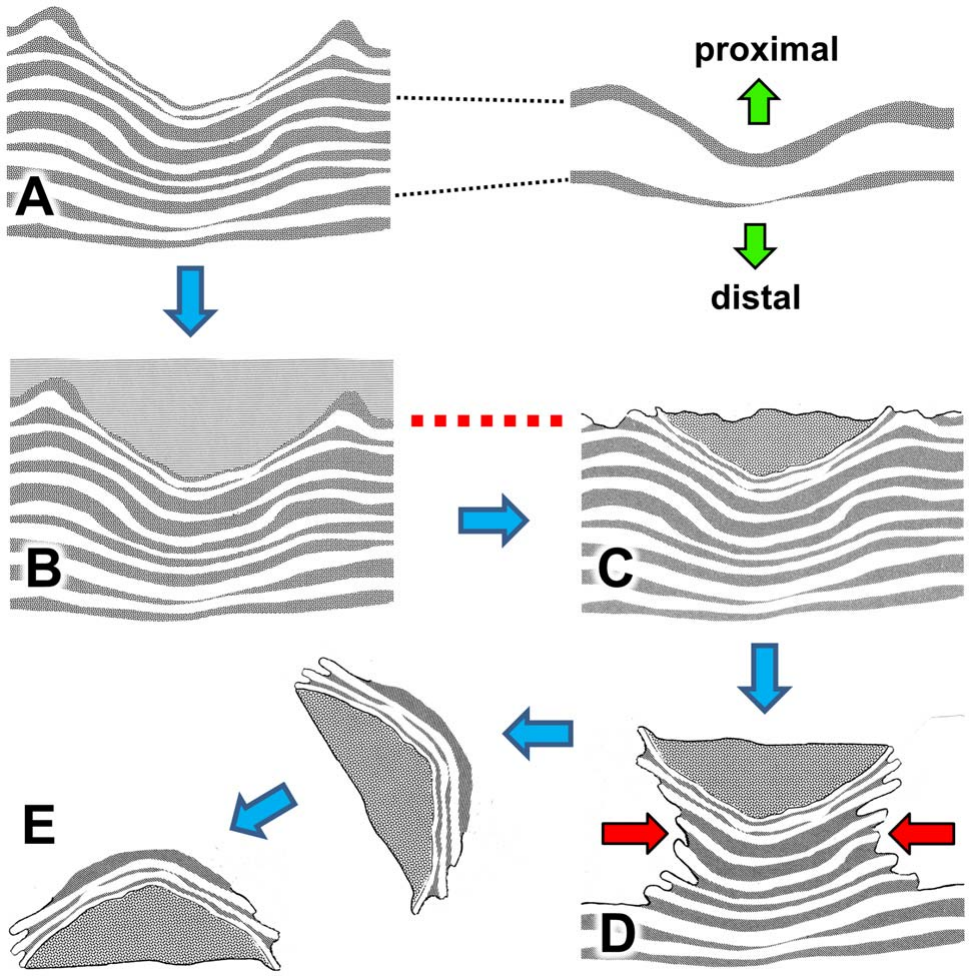
Transmitted reliefs and their effects on the preservation and appearance of sauropod footprints. A, vertical section of thinly-layered substrate showing transmitted reliefs stacked beneath a sauropod footprint (natural mould). B, same example buried by influx of sediment which fills the footprint to form its natural cast. C, following lithification (transformation of soft wet sediments to hard dry rock), erosion to level of dotted line will produce the ‘onion-ring’ pattern which is characteristic of many sauropod footprints in the Broome Sandstone - a remnant of the natural cast encircled by the eroded edges of transmitted reliefs (Figures 3,15). D, the natural cast proves more durable than the surrounding rock, which is removed by erosion to leave a rock pillar - a stack of transmitted reliefs capped and protected by a remnant of the natural cast (Figures 5B,C and 21). Further erosion undercuts the upper part of the stack (red arrows). E, the upper part of the rock pillar breaks free and is rolled over by wave action, finally coming to rest on the beach as a ‘turtle-back’ boulder (Figures 18C,D).

Some of the most eye-catching features of the Broome Sandstone are the zones of contorted bedding that underlie and surround the sauropod footprints. These create a distinctive banding or onion-ring effect wherever the rock enclosing the footprint happens to be exposed by breakage (e.g. Figures 3,15). The combination of ponderous sauropods and lagoonal environments floored by thinly-bedded sheets of sand and muddy silt appears to have been ideal for the development and preservation of these structures (Figure 16). In some places these stacks of contorted sedimentary layers are nearly a metre deep (maximum recorded to date 96 cm), though their original extent, before compaction and consolidation of the substrate, must have been considerably greater.

**Figure 17:**
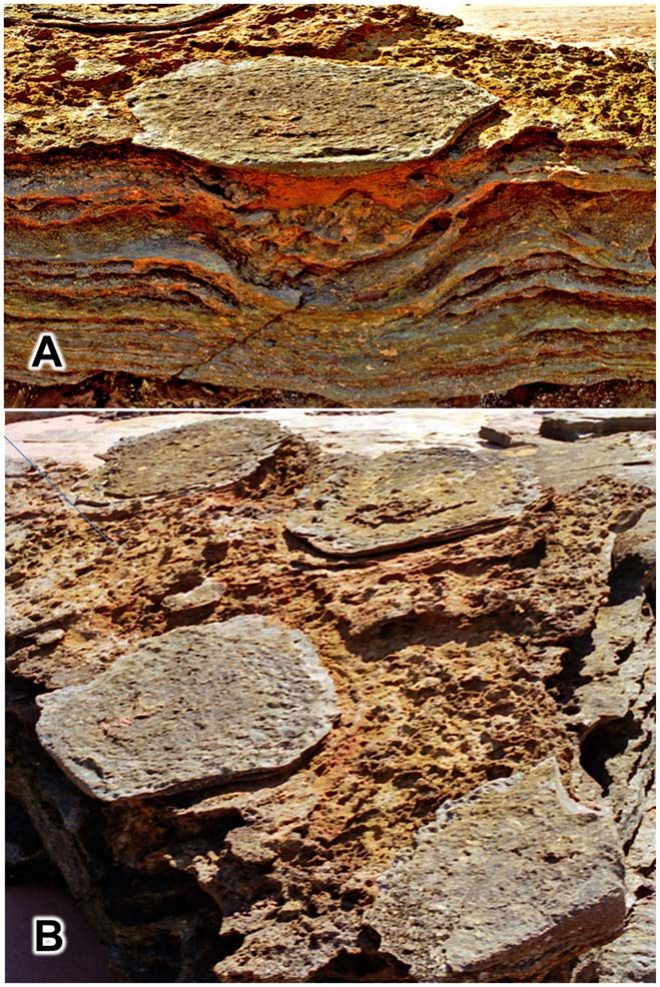
Sauropod tracks showing extent of transmitted reliefs and effects of erosion. A, near-vertical section through a sauropod pes print with stack of transmitted reliefs beneath it. As this section is through the comparatively shallow lateral part of footprint, it does not reveal the maximum depth of the transmitted reliefs (which lie beneath the deeper medial part of the print). The erosion-resistant capping of ironstone is 61 cm long. B, part of trackway containing the same footprint (at centre left), viewed obliquely from above. Note how durable ironstone capping has protected the footprints while the intervening areas of rock have been eroded more rapidly.

Here it is necessary to clarify the terminology. The contorted bands of sediment that underlie and surround the footprint have been identified in the past by a variety of names: transmitted prints, underprints, undertracks, sub-traces and ghost prints. Some of those terms lack precise definition [22], and some have been used so indiscriminately that they will certainly invite misunderstandings. For instance, the term underprint has been applied to at least three different types of biogenic sedimentary structure in the Broome Sandstone [33], [34], including that shown here in Figure 8B (which, if anything, is an overprint; see Figure 9). For the purposes of this inquiry the term footprint (print or, more loosely, track) has been defined so narrowly that it cannot be applied to any sub-surface feature. Consequently the internested layers of contorted rock that underlie the footprint (*sensu stricto*) will be identified here as *transmitted reliefs*. That term seems preferable to any other because it is both accurate and informative. The structures in question reproduce the relief or topography of the footprint (as in a relief map or *bas-relief*), though they are not footprints *sensu stricto*, and they are formed by the force of the foot’s impact being transmitted into the substrate (Figures 16,17). The significance of that terminology will be examined more closely at a later point.

The number of reliefs in the stack will express the number of detectable discontinuities in the substrate (i.e. interfaces between layers with different colour, grain-size or resistance to erosion), and in the case of the Broome Sandstone these happen to be unusually numerous: in places the individual layers of sandstone and siltstone are only a few millimetres thick (e.g. Figure 15). The number of discontinuities (or the thinness of the bedding) is important in controlling the development and the visibility of transmitted reliefs. A thinly-bedded substrate reveals the existence of transmitted reliefs with great clarity, whereas a substrate of thicker and more homogeneous beds would betray little or nothing of their existence. In that respect the frequency of discontinuities is (inversely) analogous to the contour interval on a topographic map.

**Figure 18:**
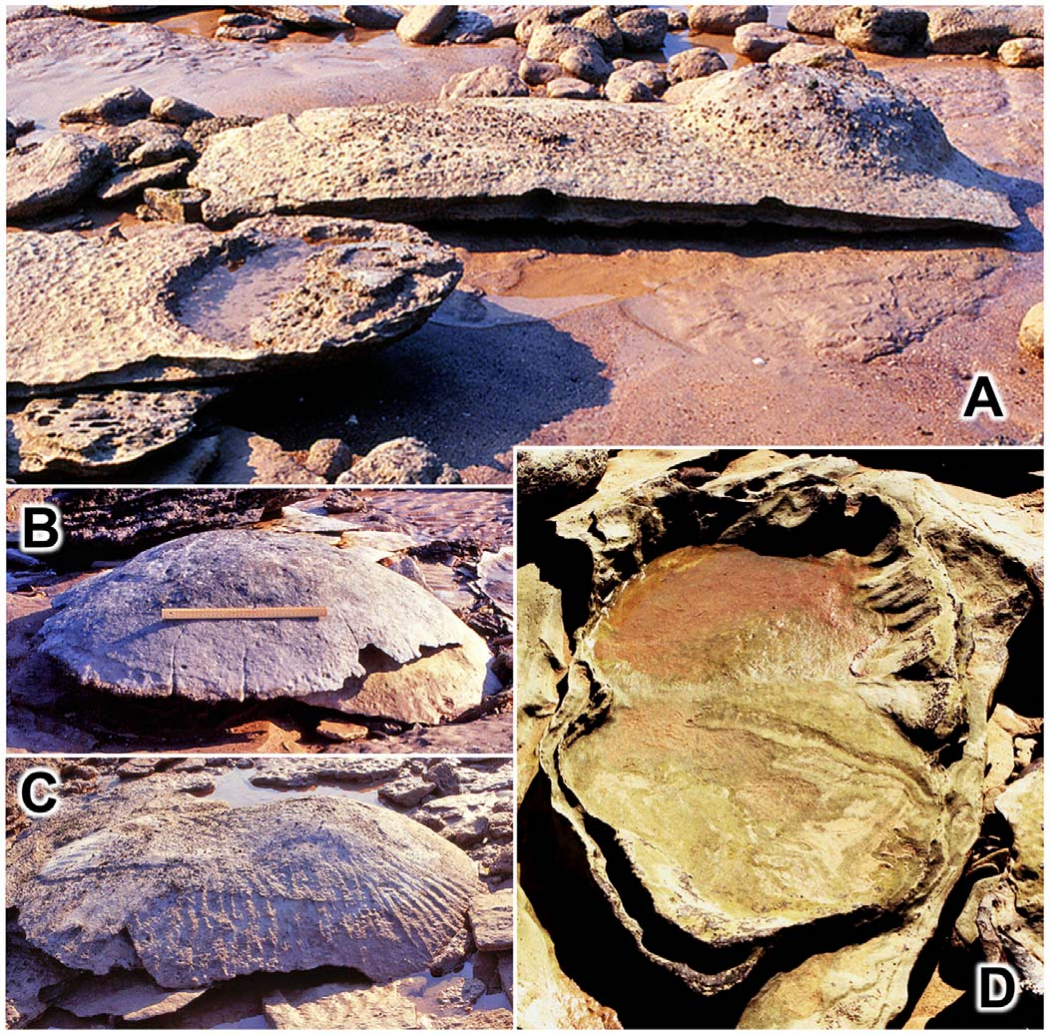
Transmitted reliefs: some examples of unusual appearance and preservation. A, thin sheets of sandstone moulded into the form of transmitted reliefs and exposed by natural erosion. The long sheet of sandstone in the background is a fragment of transmitted relief from a large basin-like feature (Figures 20–22); it has been rolled over by wave action to expose its convex sole and one very conspicuous transmitted relief of a single sauropod pes print. B, a characteristic turtle-back boulder - the core of a durable footprint cast, encased in transmitted reliefs, which has been freed by erosion and come to rest on the beach. Scale is 1 foot (c. 31 cm). C, a double turtle-back boulder comprising the cores of two footprint casts. D, a basin of transmitted relief with figure-8 outline; derived from the stacks of transmitted reliefs below two near-coincident footprints.

The fidelity of any given relief (i.e. the extent to which it reproduces the relief of the footprint *sensu stricto*) is governed by a combination of factors. These include the physical properties of the sediment at that particular horizon in the substrate and the extent to which other layers in the substrate might impede or reflect the transmission of forces generated by the foot’s impact. In general the fidelity of relief declines from proximal (immediately beneath the footprint) to distal (at the deepest level in the substrate and most remote from the footprint; Figure 16A). In some instances a proximal relief reproduces the overlying footprint with such fidelity that it might be treated as a duplicate or mistaken for the footprint itself. In fact, some proximal reliefs may preserve more and finer detail than the footprint. This may happen, for example, when details of the footprint’s topography are obliterated by slumping or smearing on withdrawal of the foot but are transmitted to a slightly deeper level and preserved there. At the other extreme the distal reliefs are little more than vague dish-like features with no special resemblance to the footprint. Each layer of rock detected in the stack beneath the footprint has two reliefs (Figure 18A) which are complementary to overlying and underlying reliefs: its proximal surface presents a concave epirelief, corresponding to the topography of the footprint *sensu stricto* (a natural mould), and its distal surface is a convex hyporelief, corresponding to the topography of the sole of the footprint’s natural filling (or cast).

In some parts of the Broome Sandstone sauropod footprints (or those parts of the transmitted reliefs directly beneath the footprints) are preserved on pedestals whereas the surrounding rock is removed more rapidly by erosion (e.g. Figures 5B,C). In some instances, at least, those rock pedestals have been protected by a cap or plug of durable sandstone representing the footprint’s natural filling (Figure 16D,17). However it is also possible that some pedestals that lack any trace of such a protective cap might owe their existence to enhanced induration of the rock layers underlying the footprint. The zone of transmitted reliefs that lies directly beneath the footprint is compacted twice - first by the impact of the track-maker’s foot and, then, a second time during the complex process of lithification (the transformation of soft wet sediment into dense hard rock, involving compaction, consolidation and cementation). By contrast those parts of the transmitted reliefs that lie outside the ambit of the footprint are compacted only once, during lithification. It is conceivable that double compaction might have enhanced the durability of the rock column directly beneath the footprint. However, in the current state of knowledge it is difficult to determine whether some stacks of transmitted relief persist in the form of pillars because they really have been indurated by double compaction or whether they survive because they once possessed durable cap-stones, now lost to erosion.

The durable vestiges of sauropod tracks supported on pedestals are eventually undercut by erosion (Figure 16D), detached and rolled over in the surf (Figure 16E), finally coming to rest on the beach as distinctive turtle-back or whale-back boulders (Figures 18B,C). Although these objects are easily mistaken for water-worn boulders, they are in fact the fillings (natural casts) of sauropod dinosaur tracks encased in remnants of the more proximal transmitted reliefs.

### Multiple footfalls

**Figure 19:**
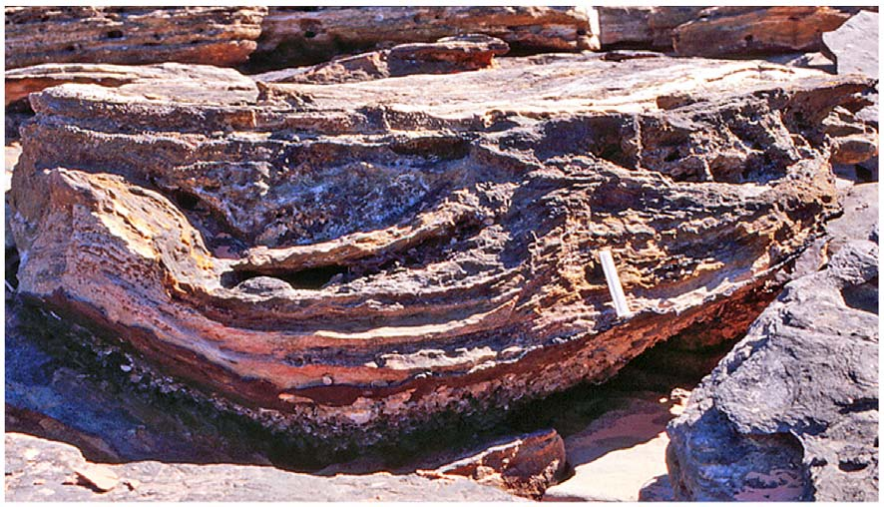
Hierarchy of transmitted reliefs: the basic elements. Two sauropod footprints, each underlain by its own stack of transmitted reliefs, are enclosed in a single larger basin of transmitted reliefs. Scale is 1 foot (c. 31 cm), but tilted and foreshortened. This specimen encapsulates the basis of hierarchical pattern - two stacks of transmitted reliefs nested into a single larger basin of transmitted reliefs.

In the standard walking gait of a sauropod [38], [42], [43] each hindfoot was planted on or near the newly-formed print of the ipsilateral forefoot. Thus, there were two nearly coincident footfalls in rapid succession, forefoot then hindfoot, and the effect of this coupling would have been cumulative or, if sufficiently rapid, synergistic. The substrate might be softened or liquefied by the impact of the forefoot before sustaining the impact of the hindfoot. The same effect might result from any two coincident or near-coincident footfalls, whether from a single animal or from two. The outcome of this double impact was a composite feature of deformation, where the stacks of transmitted reliefs formed individually by the two footfalls are enclosed jointly in a bigger and deeper basin of deformation (Figure 19). In the terminology used here, that larger basin is a stack of transmitted reliefs from a manus-pes couple or from two near-coincident footfalls. The existence of such a composite feature, the basis of a hierarchical pattern, seems never to have been reported previously.

**Figure 20:**
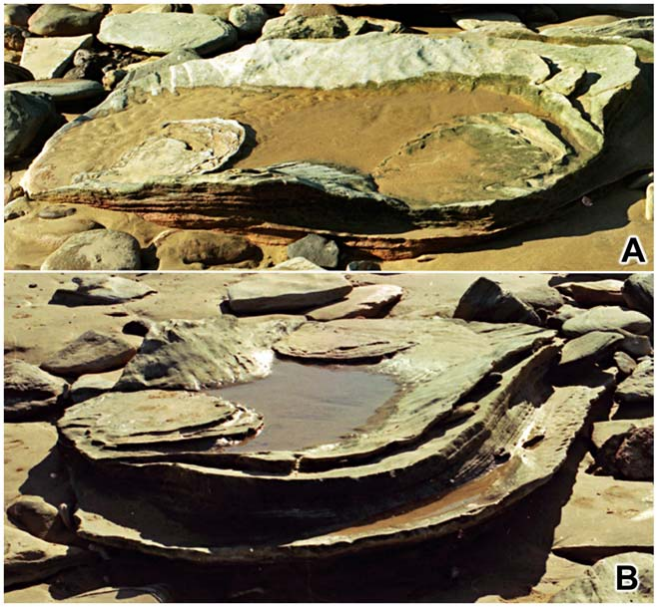
Hierarchy of transmitted reliefs: a saddle-shaped basin. A,B, two views of single saddle-shaped basin of deformation containing residual stacks of transmitted reliefs from two sauropod footprints. The two photographs were taken on different occasions and in the interim a storm removed some of the obscuring beach sand.

**Figure 21 pone-0036208-g021:**
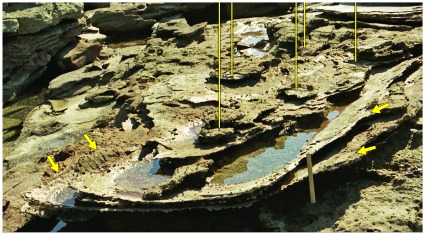
Hierarchy of transmitted reliefs: an entire sauropod trackway. A trough of deformed substrate extending from upper right to lower left betrays the route taken by a sauropod dinosaur. Arrows indicate the steeply dipping flanks of the trough; vertical pointers identify much-eroded stacks of transmitted reliefs representing individual pes prints. Scale indicated by 1 ft (c. 31 cm) ruler at lower right. This complex pattern of substrate deformation cannot be detected by conventional search for pristine (‘museum-grade’) footprints on an intact bedding plane; it is revealed only in broken and eroded specimens which are often deemed to be of inferior quality.

Such basin-like features tend to be roughly circular or elliptical in plan, but in addition there are some more elongate and trough-like patterns of deformed substrate. In these a single slow-walking sauropod sometimes planted one hindfoot sufficiently close to the print of its other hindfoot for the two resulting stacks of transmitted reliefs to coalesce into a single basin of deformation which is saddle-shaped (Figure 20), with the outline of a figure 8 in plan view (Figure 18D). Examples detached by erosion may be found on the beach as turtle-back boulders with two domes, rather than one, and an appropriate figure-8 outline (Figure 18C). A chain of these saddle-shaped basins would then unite to form an even bigger and deeper-lying trough marking the animal’s line of progress (Figure 21). In this manner a single walking sauropod could deform an evenly layered substrate into an elaborate hierarchical structure, with small stacks of transmitted reliefs nested into successively larger stacks of basins which, in turn. are nested into a single trough.

These complex hierarchical patterns of substrate deformation appear to be unprecedented. Their existence cannot be detected by inspecting pristine footprints on an intact bedding plane, but only by investigating the broken and eroded specimens which are usually assumed to be of inferior quality. Consequently it is not known whether the hierarchical patterns of deformation detected in the Broome Sandstone might occur at any other sauropod track-sites: their existence has never been suspected, and no one has ever searched for them.

### Large-scale effects

**Figure 22:**
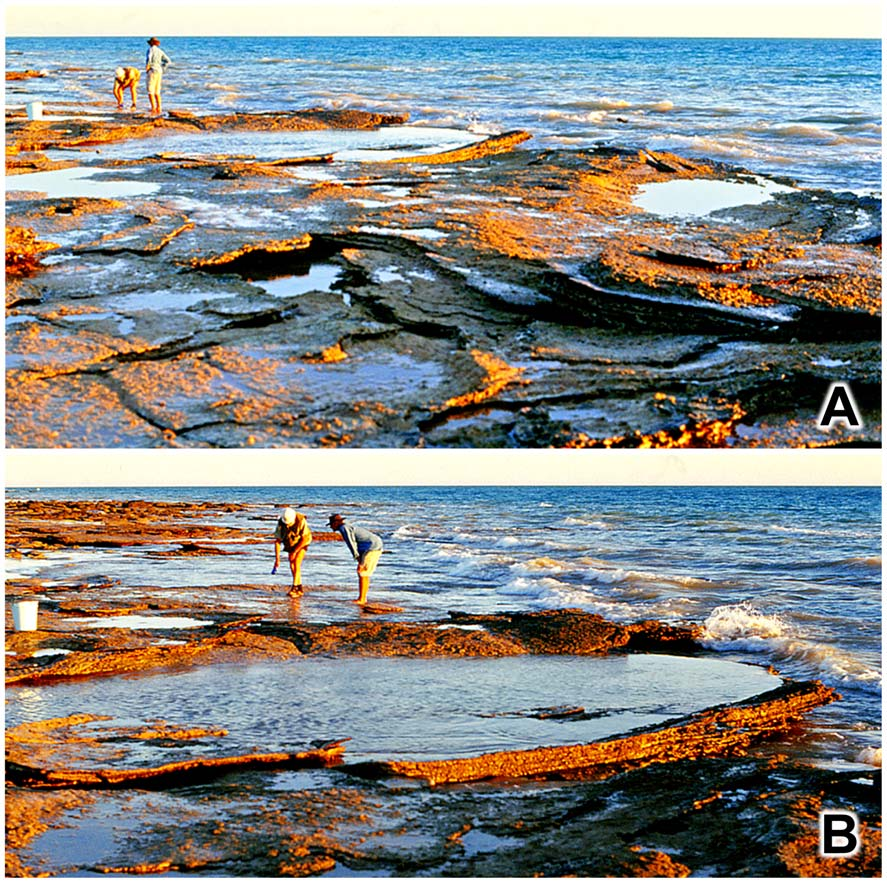
Basins and channels produced by the impact of sauropod feet. A, short stretch of coast with evidence of much sauropod traffic; along the seaward margin two large basins, resembling shallow synclines, are separated by an eroded area resembling a minor anticline. B, closer view of the larger basin shown above; note remnants of distinct sauropod footprints at extreme left.

At various sites around the world sauropod tracks have been found in natural aggregations, often with parallel trends (e.g. [44]) indicating that the track-makers may have travelled in groups along preferred routes. Similar aggregations are evident in the Broome Sandstone, with predictable consequences for deformation of the substrate. Since two near-coincident footfalls could interact to deform the substrate into a single basin (Figure 19), it is not surprising to discover that increasingly large numbers of near-coincident footfalls could interact to produce even bigger and bigger basins of deformed substrate (e.g. Figure 22).

**Figure 23:**
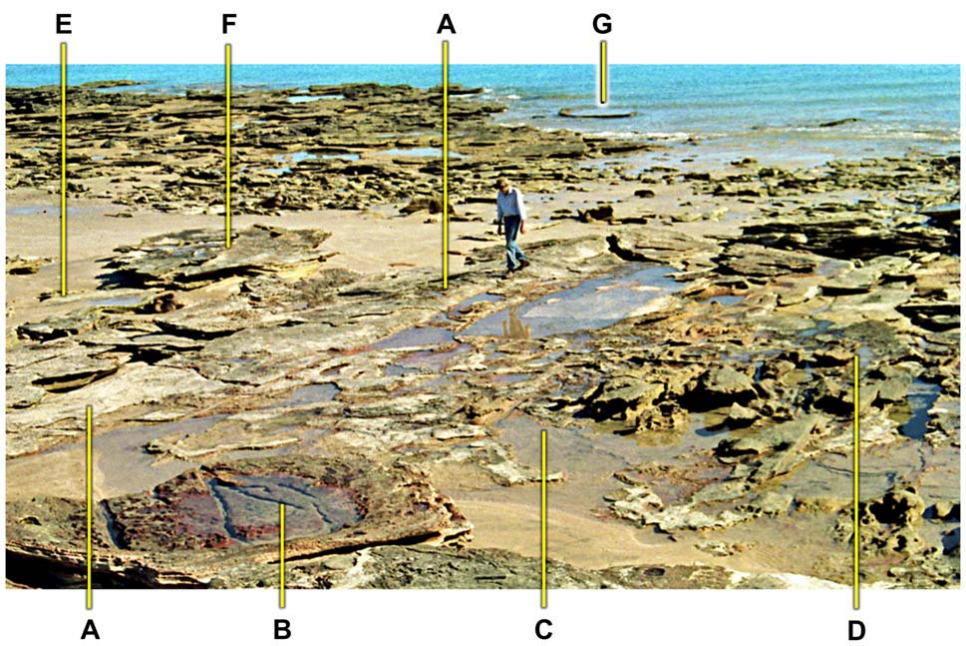
Part of shore platform viewed from cliff-top at low tide. A, crest of a ‘monoclinal’ fold flanking a trough trodden down by sauropod dinosaurs (near side, with remnants of numerous footprints among the puddles of sea-water); beds on far side of the crest are folded down to a lower level than those on the near side. B, crater-like area of subsidence shown in Figure 13. C–D, area trodden by sauropods (but largely concealed by rubble); there is no definite border corresponding to A–A. E, a smaller ‘monoclinal’ fold, with correspondingly small trough to near side. F, wide but shallow basin containing sauropod tracks (resembling that in Figure 22, but broken into two parts). G, another basin with sauropod tracks, about to be inundated by the rising tide.

At some sites the parallel and closely-grouped troughs produced by two or more sauropods have merged into even more extensive channels and troughs. These enormous stretches of deformed substrate resemble minor synclinal folds (Figures 21,22) and are frequently several metres wide and up to 20 metres or more in length. They are, in fact, dinosaurian thoroughfares flanked by areas of untrodden substrate resembling asymmetrical anticlines or monoclines (e.g. Figure 23). The flanks of these miniature folds may dip at an angle of 60° or even more, in striking contrast to the regional dip of only 2–3°. Beyond a length of 20–25 metres these very large trough-like features tend to dissolve into individual trackways, scattered footprints and ill-defined areas of trampled substrate. What may appear at first glance to be a reasonably well-defined channel or thoroughfare invariably proves to be open-ended or to have only a single well-marked flank that can be traced for more than 20 metres or so.

**Figure 24:**
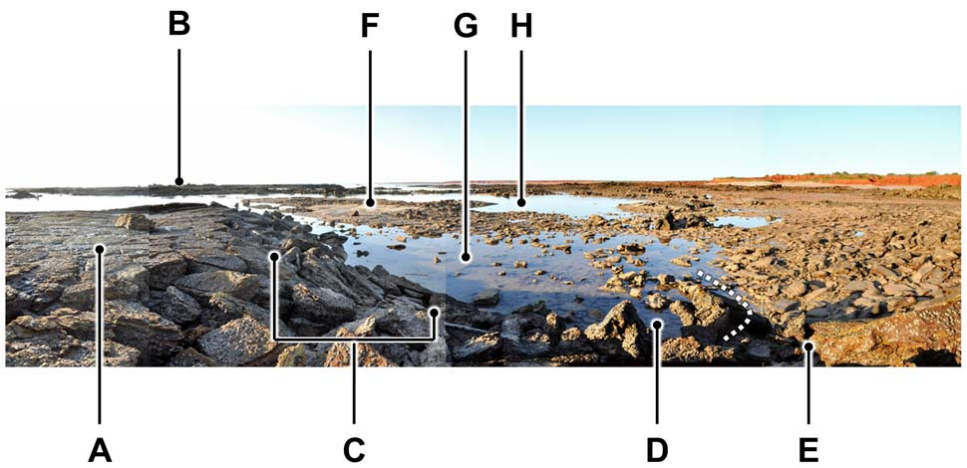
Composite panorama of James Price Point, about 60 km north of Broome. Looking northwards from the southern side, at evening low tide, 18∶46, July 24 2009. A, elevated area of flat-lying beds, trodden only by small bipedal dinosaurs (see Figure 25), not by sauropods. B, corresponding elevated area. C, concave sloping flank of the low-lying area trodden down by sauropod dinosaurs; the slope has been slightly exaggerated by erosional undercutting, collapse and slipping, but is nonetheless the curved flank of a basin or trough (see Figure 27). D, arcuate end of a basin (strike of the bedding indicated by dotted line). E, end of one long water-filled channel (extending from mid-left) representing a thoroughfare or heavily trampled route used by sauropods; the landward part of shore is obscured by rubble and sand. F, flat-lying beds exposed at the core of a low-amplitude ‘anticlinal’ fold which intervenes between the water-filled channels G and H; this represents a less-heavily trodden area between two major dinosaurian thoroughfares. Although the terrain has been somewhat reduced by modern erosion, it still conveys a reasonably faithful impression of the Early Cretaceous topography.

**Figure 25:**
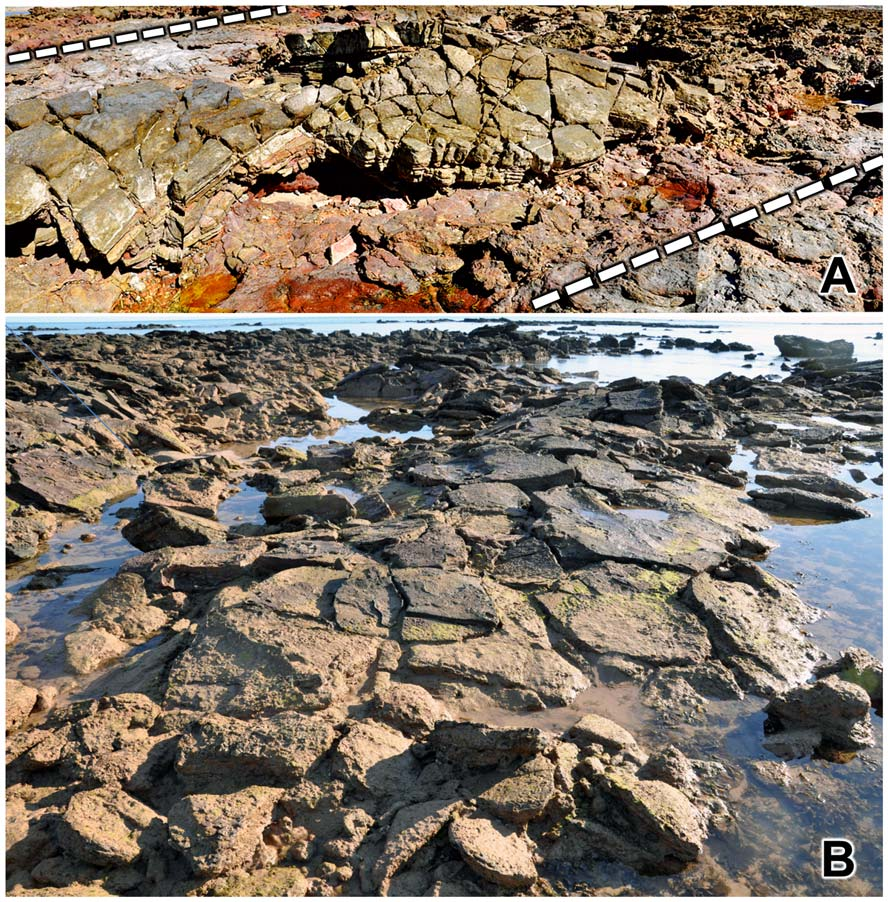
Thoroughfares and troughs produced by sauropod dinosaurs. A, residual hummock or ‘anticlinal’ fold of lagoonal sediments lying between two dinosaurian thoroughfares (with axes indicated by dashed lines). The thoroughfares are so deeply trodden that they have exposed the underlying beds - red palaeosols (weathered grey) with vestiges of sauropod tracks; south of James Price Point. B, a similar but smaller feature at James Price Point, at the very margin of the lower-lying areas shown in Figure 24. The two water-filled areas at left and right have been trodden down by sauropods to leave an ‘anticlinal’ fold between them.

**Figure 26:**
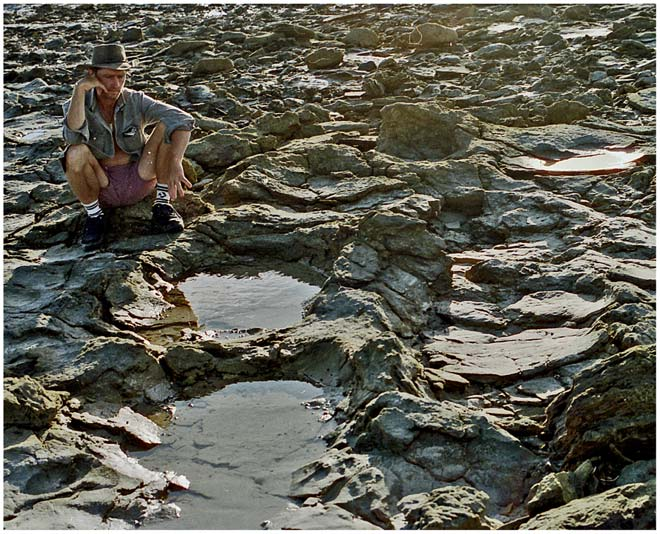
The middle of a dinosaurian thoroughfare, thoroughly trampled by sauropods. Examples such as these, to the south of James Price Point, tend to be ephemeral, as the thinly-bedded rock is rapidly stripped away and broken up during the annual cyclone season. A few moderately large (30–35 cm) three-toed tracks of predaceous theropod dinosaurs (ichnogenus *Megalosauropus*) have been found in these severely trampled areas, but the somewhat smaller three-toed tracks of plant-eating ornithopod dinosaurs (e.g. ichnogenus *Wintonopus*, in Figure 28) appear to be completely absent.

**Figure 27:**
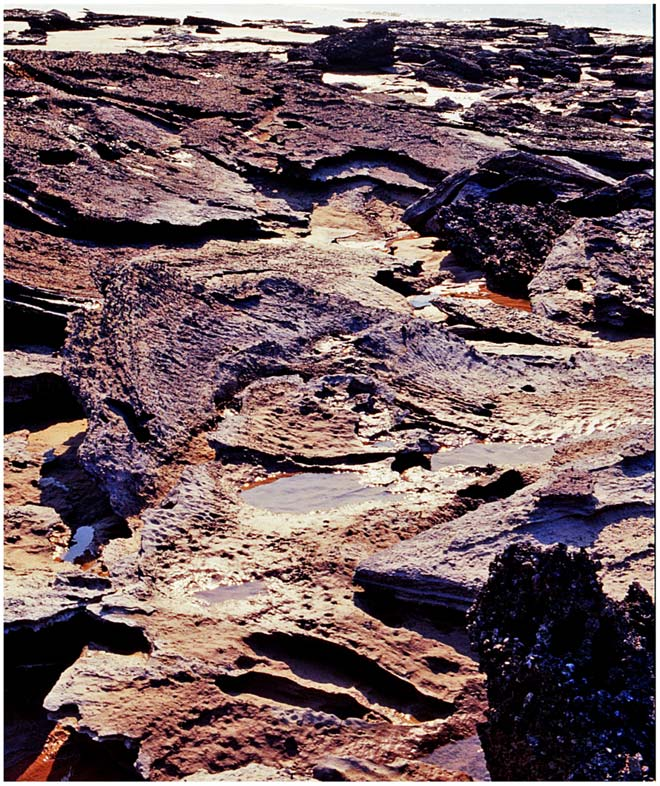
The curved flank of a dinosaurian thoroughfare. The area shown here is at the margin of the elevated region A in Figure 24. Transmitted reliefs of sauropod tracks are visible in foreground.

**Figure 28:**
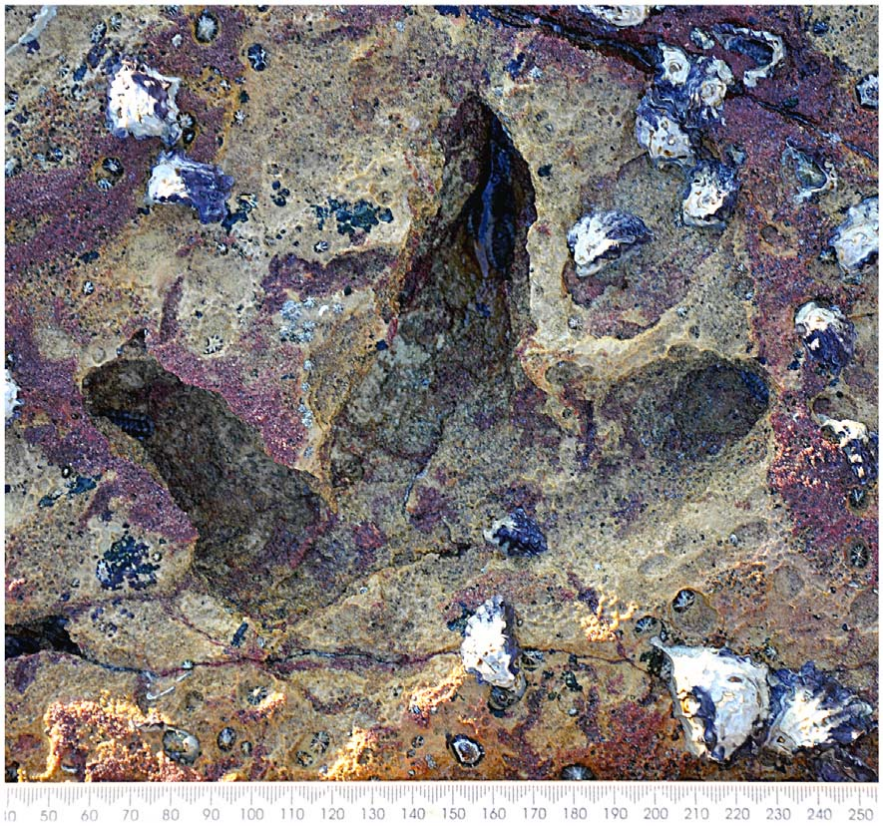
Left pes print of small ornithopod dinosaur, cf. ichnogenus *Wintonopus*. Tracks of this type are found on the elevated areas of the shore at James Price Point (e.g. A,B in Figure 24), but not in the lower-lying areas that were trodden by sauropods. It is tempting to suppose that these smaller dinosaurs preferred higher ground, thereby avoiding the heavy traffic of sauropods.

The effects of sauropod trampling are seen on the grand scale at James Price Point, about 60 km north of Broome (Figure 24). Although the scene appears at first glance to be little more than a field of rubble, it is actually an Early Cretaceous landscape which has been preserved more or less intact and is currently being exhumed by coastal erosion. The deepest areas of the shore, visible at extreme low tide as long water-filled channels, are the axes of dinosaurian thoroughfares, so intensively trampled that in places they expose inliers of the underlying beds (cf. Figure 25A). Despite the severe trampling of such areas it is still possible to detect remnants of individual sauropod tracks (Figure 26), though these are ephemeral and rarely survive the vicissitudes of the annual cyclone season. Along the less intensively trampled margins of those thoroughfares it is much easier to detect individual sauropod tracks and remnants of discrete basins and channels of deformed substrate (e.g. Figure 25B). Then, above the curved and steeply-dipping flanks of those channels and basins (Figure 27), there are still ridges and outliers of the undisturbed lagoonal substrate, sometimes marked with the tracks of small bipedal dinosaurs (e.g. Figure 28). James Price Point may be the only place on Earth where one may gaze out over an Early Cretaceous landscape that has been extensively reshaped by the everyday comings and goings of sauropod dinosaurs.

## Discussion

### Definitions and taxonomic practice

The term footprint (or print) has been used throughout in a narrow sense to identify that area of the substrate moulded by direct contact with the underside of the track-maker’s foot. A transmitted relief replicates the topography of that footprint to some (usually limited) extent but is not, itself, a footprint *sensu stricto*. That distinction between footprint and transmitted relief might seem to express nothing more than personal preference in the choice of terms, for there is clearly a similar distinction between the direct and indirect tracks of Gatesy [35], the tracks and underprints (*sic*) of Lockley [45] and the tracks and undertracks of many subsequent authors (e.g. Milàn et al. [37]). However, the terms used here are something more than convenient labels: they are intended to dispel the ambiguities that pervade existing terminology.

Although it is tempting to describe a transmitted relief as a subdued or muted version of the footprint, the two structures are fundamentally different. The footprint is impressed directly by the track-maker into a surface exposed to the air or covered by water, whereas a transmitted relief has no contact with the track-maker and is formed beneath a blanket of sediment. In fact, the track-maker has projected an indication of its existence and its activity (initially a trace and potentially a trace-fossil) into sediment that was deposited and buried before the animal arrived on the scene - and conceivably before the track-maker ever existed. Transmitted reliefs are intrusive elements projected into sedimentary deposits of the past, the very antithesis of derived fossils, whereas the footprint (*sensu stricto*) must be contemporary with the animal that made it. Likewise the natural cast is fundamentally different in nature from the corresponding reliefs transmitted into the substrate. The natural cast is formed after the track-maker has impressed its footprint and departed from the scene, whereas the transmitted reliefs are formed at nearly the same instant as the footprint and are composed of sedimentary material that was already *in situ*.

Unfortunately those distinctions are not acknowledged in the prevailing terminology, which is dominated by the term track. Aside from occasional reference to overtracks (e.g. by Marty [46]), ichnological literature currently maintains that tracks exist in two forms - (1) true or direct tracks, and (2) undertracks or indirect tracks (with several synonyms). It seems to be agreed universally that the objects in the second category (whatever you might choose to call them) are *not* true tracks, and they would not normally be accepted as an adequate basis for defining ichnotaxa. Consequently their status is unclear: they seem to be regarded as tracks of some sort, though they are excluded from the classification of ‘true’ tracks.

Their status may be clarified by considering their origin. What has been transmitted into the substrate beneath a footprint (*sensu stricto*) is not a footprint or a track of any kind: it is the force of the foot’s impact. And the transmitted force has interacted with existing sub-surface structures (laminations) to replicate some physical characteristics of the footprint (size, shape and topographic relief), though only approximately and to a limited degree. Even so, the term under*track* (or under*print* [45]) certainly seems to imply that tracks of some sort may be transmitted into the substrate. Yet, at the same time and in a broader context, it is generally agreed that tracks of any kind must be autochthonous fossils. The remnants of a track-maker’s carcass may be transported into an alien environment and preserved there in the form of body fossils, but its tracks cannot be transported in the same manner. Indeed, the scientific value of fossil tracks resides largely in the fact that they are *not* transportable: they are, for that very reason, the most significant and trustworthy clues to the geographic distribution and habitat preferences of ancient track-makers. Now, if tracks cannot be transferred horizontally, from one geographic setting to another, it would seem even less likely that they could be transmitted or transported vertically, from one stratigraphic horizon to another. In that case the sub-surface features called undertracks could not be tracks of any description. In short, the common distinction between tracks and undertracks seems to skirt round an inconsistency: it acknowledges that tracks cannot be transported horizontally but suggests that they are transported vertically.

All that confusion and uncertainty stems from indiscriminate use of the word track. In any given context that all-embracing term might refer to anything from a single footprint (*sensu stricto*) to an entire dinosaurian thoroughfare, including objects which are declared to be something other than true tracks and are denied the formal status of tracks (in the sense of ichnotaxa). It is difficult to imagine a more confusing system of terminology.

The terms introduced here will permit escape from the existing paradox, in which tracks (*sensu latissimo*) are held to comprise true tracks and tracks which are *not* true tracks (i.e. undertracks and overtracks). The term *footprints* refers explicitly and unambiguously to true or direct tracks, whether singly or in natural groups (manus-pes couples and trackways); and the term *transmitted reliefs* (*of footprints*) will distinguish undertracks or indirect tracks. Here the word relief is used in its conventional sense for an object showing elevation or projection from a plane surface, as in a relief map or *bas-relief*. The minor features called overtracks (*sensu* Marty [46], not ‘overprints’ *sensu* Lockley [45]) hardly warrant a special designation; they are surely no more significant than those that might happen to overlie ripples, pebbles, erosional features or other irregularities of the surface traversed by a track-maker. The word *tracks* is retained only as a generic term.

That terminology acknowledges that footprints and transmitted reliefs are fundamentally different structures, and in view of their profound differences it seems reasonable to exclude transmitted reliefs and other adventitious features from the realm of ichnotaxonomy. If transmitted reliefs were admitted to be footprints, or structures equivalent to footprints, they might be referred to an existing ichnotaxon or used in erecting a new ichnotaxon. Logically the same concession should extend to the basin-like features which are the transmitted reliefs of a manus-pes couple, and then to the trough-like features which are the transmitted reliefs of an entire trackway. In theory the same concession might extend ultimately to regions of deformed bedding that resemble minor tectonic structures and even to the larger features of physical geography seen at James Price Point. In effect, the state of ichnotaxonomy would come to resemble that of zoological taxonomy when the available names of taxa were extended to the ‘work’ of animals [47]. Seemingly valid ichnotaxonomic names might be bestowed on geographic features of the Dampier coast, in just the way that the name *Homo sapiens* might be applied to all and any human artefacts, from stone axes to space shuttles.

It seems preferable to avoid that incongruous outcome by maintaining the genuine, if arbitrary, distinction between footprints and sedimentary structures (patterns of deformation) which are *associated* with footprints. That policy is, in fact, consistent with conventional practice in ichnotaxonomy, where features of transmitted relief are disregarded or treated, at best, as an indirect and inferior source of information about the ‘true’ footprints. Footprints, *sensu stricto*, are definitely objects of organic origin whereas the development of transmitted reliefs depends as much on the nature of the substrate as it does on the intervention of a track-maker. In fact, the development of transmitted relief, in the broadest sense, does not *necessarily* require the active involvement of a track-maker. In theory transmitted reliefs might be produced by organisms which are inert (e.g. a carcass settling on to the floor of a lagoon) or by the impact of inorganic objects such as drop-stones, lapilli, volcanic bombs, meteorites or hail.

Even so, the taxonomic implications should not be overrated. Ideally ichnotaxa should be established on type material comprising one or more footprints (true tracks), not transmitted reliefs (undertracks). But that is merely the description of ideal practice; it is not the stipulation of a mandatory requirement. Each case is to be judged on its individual merits, and no great harm will ensue if a valid ichnospecies should transpire to be founded on transmitted relief rather than a footprint (a true track). In practice all that matters is that type material should be adequate and diagnostic, regardless of its status as footprint or transmitted relief. That concession is not the thin end of a wedge that would ultimately permit all and any transmitted reliefs to be classified as conventional ichnotaxa, because only the most proximal reliefs are likely to retain the morphological details required to discriminate a valid ichnospecies. The more distal transmitted reliefs lack such consistent morphological detail and are far less likely to be mistaken for footprints (true tracks) - though they might easily and more appropriately be classified as a series of sedimentary structures (e.g. bowls, basins, troughs and folds of various shapes and sizes).

### Previous interpretations

Some of the sedimentary features described here may have attracted attention in the past, though the sauropod tracks were not explicitly identified as such until the 1990s. A brief report on the geology of James Price Point [32] noted areas of convoluted bedding in the Broome Sandstone, but was unable to explain their origin. It suggested that these perplexing features might be the ‘crawlways’ of giant Cretaceous turtles, though the example that was illustrated ([32], figure 4) bears strong resemblance to some of the transmitted reliefs which are so commonly associated with the sauropod tracks (e.g. at lower right of Figure 26).

Two brief reports on the geology and palaeontology of the same stretch of coast [33], [34] were somewhat contradictory and decidedly noncommittal. Throughout them the term underprint was applied indiscriminately to as many as three different patterns of sedimentary structure, of which only one (or, perhaps, two) would agree with the concept of transmitted relief used here. The first of those reports noted that sauropod tracks were relatively abundant but also maintained that many of them would probably transpire to be potholes. However, some of the examples that were illustrated ([33], figure 1, foreground] show all the defining characteristics of sauropod tracks, including the shallow kidney-shaped manus prints and the impressions of broad flat claws curving around the outer rim of the much bigger pes prints. Indeed, some of those specimens might even qualify as textbook examples of sauropod tracks, and they are definitely not potholes. The second report [34] was even more circumspect and referred to the sauropod tracks only as ‘putative sauropod underprints’ or ‘circular structures’. It went on to suggest that they might be cavities left by sandstone casts of tree-stumps or the feeding-traces of sting-rays. Neither of those possibilities will bear close scrutiny: they are, in fact, two fairly common misinterpretations of dinosaur tracks, both mentioned elsewhere [22] in a brief survey of similar misconceptions.

**Figure 29:**
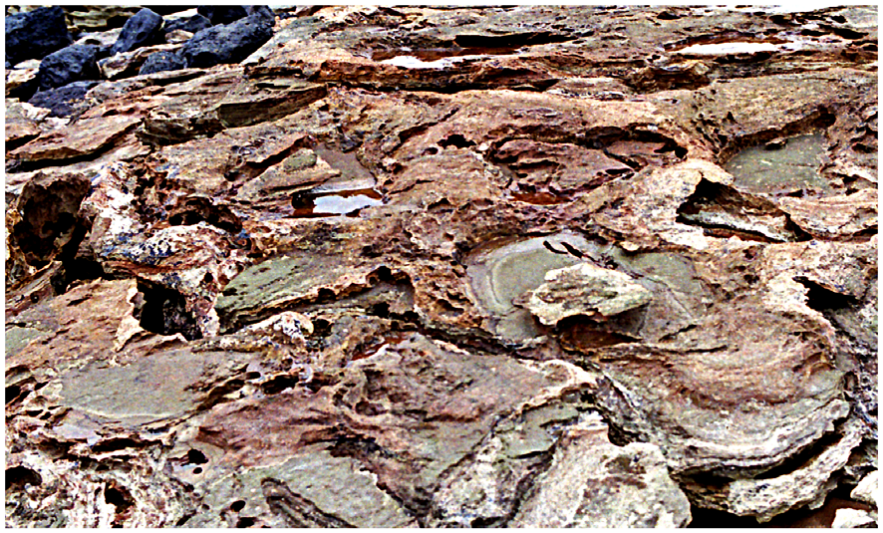
Crumpled bedding - the result of trampling by sauropods. Previous reports of contorted bedding in the Broome Sandstone may well be based on similar occurrences. Individual sauropod footprints are still discernible, despite the severe trampling.

At a much earlier date Brunnschweiler [48] reported on a geological reconnaissance of Carnot Bay, to the north of James Price Point, There Brunnschweiler encountered some localized areas of buckling and convolution in the otherwise flat-lying beds of the Broome Sandstone and remarked that these might easily be mistaken for minor tectonic features. Some of that convoluted bedding might well have been the product of trampling by sauropods, as is certainly the case at other sites along the Dampier coast (e.g. Figure 29). However, Brunnschweiler drew particular attention to some miniature anticlinal folds or domes, which he described as ‘blisters’, and speculated that these might have been forced upwards by the subaqueous swelling of clay minerals. If the features reported by Brunnschweiler resembled those described here, the ‘blisters’ might correspond to the untrodden areas of substrate intervening between troughs and basins formed by the passage of sauropod dinosaurs (Figure 25). The ‘blisters’ would not have been forced upwards: they would have remained *in situ* while the surrounding areas were trampled down by the comings and goings of sauropod dinosaurs.

Brunnschweiler [48] made no mention of sauropod or any other dinosaur tracks, but that omission is not significant, as their existence was unknown at the time of his reconnaissance. Before the 1990s there were very few reports of dinosaur tracks in the Broome Sandstone [1], [11], [49], and these referred only to three-toed footprints, in line with the popular belief that dinosaur tracks should resemble gigantic bird tracks. The existence of the far more abundant sauropod tracks was not reported until the 1990s, for the simple reason that these went unrecognized. In 1964, for instance, E.H. Colbert - at that date the world’s foremost authority on dinosaurs - examined the three-toed tracks known to occur at Gantheaume Point, near Broome [49], but neither he nor any of his companions noticed the existence of sauropod tracks at the same site, sometimes less than a metre away from the three-toed tracks that occupied their attention. (In fairness it must be added that the sauropod tracks at Gantheaume Point are very poorly preserved and are still overlooked by visitors at the present day.) Even if Brunnschweiler had encountered sauropod tracks at Carnot Bay, it is unlikely that he would have recognized their true identity, let alone their possible connection to his troublesome ‘blisters’.

### Distribution

Many of the structures described and illustrated here, such as the marginal rim of displaced sediment and the transmitted reliefs, are known to occur in association with dinosaur tracks elsewhere in the world, though the examples in the Broome Sandstone are sometimes developed to a degree that seems unprecedented. Basic understanding of such adventitious features emerged initially from direct observation of fossil footprints and their modern analogues (e.g. [35], [39], [50], [51]), though more recently there has been greater emphasis on experimental studies (e.g. [52]–[54]), sometimes with the use of artificial substrates (e.g. [55], [56]) and computer simulations (e.g. [41]). The observations presented here are generally consistent with the findings of those earlier studies, though none of them is directly comparable in every respect.

Detailed comparisons are thwarted by the scarcity of information from sauropod track-sites elsewhere in the world. Few investigators have studied sections cut through real dinosaur tracks (e.g. [37], [46], [57]), and most of those were concerned with the three-toed tracks of theropods (predaceous dinosaurs). Patterns of substrate deformation associated with sauropod tracks remain largely unexplored, though there have been some incidental observations in the quest for pristine footprints on intact bedding planes (e.g. [19], [36], [44]). However, Marty [46] has investigated vertical sections cut through two sauropod pes prints (40 cm and 43 cm long) in Late Jurassic shelf carbonate deposits in Switzerland. Both prints were fairly shallow (3 cm and 8–9 cm respectively) and were underlain by few and ill-defined layers of deformed sediment. In fact, the transmitted reliefs of those Swiss sauropod tracks were no more pronounced than those detected beneath three-toed dinosaur tracks of more modest dimensions (<25 cm long [37], [57]). That admittedly meagre evidence seems to confirm that the development of transmitted relief depends more on the physical properties of the substrate than on the size and shape of a track-maker’s foot.

Several of the features reported here, such as the hierarchical stacking of transmitted reliefs, seem to be unprecedented. Are they really unique to the Broome Sandstone? Or is it the case that similar features do occur at track-sites elsewhere in the world but have yet to be identified? While it is currently impossible to answer that question, two factors should be borne in mind.

First there is conventional practice, which is unlikely to detect the sorts of features illustrated here. Most research in dinosaurian ichnology has been focussed on the quest for morphological information, the raw material of ichnotaxonomy. The ideal research material tends to be envisaged in the form of pristine footprints or specimens of ‘museum-grade’ [32], which should supply the best information for ichnotaxonomic purposes and should, in theory, provide the most reliable clues to the identity of the track-maker. From that viewpoint adventitious features are seen essentially as distractions or imperfections and are deliberately excluded from taxonomic assessments, though transmitted reliefs might occasionally be admitted as a second-rate source of information about the morphology of true footprints. The emphasis is largely on dinosaurs rather than footprints *per se* - or, still less, on the vagaries of footprint preservation. In these circumstances it would not be surprising if some features of substrate deformation were to pass unnoticed as a matter of routine, simply because their detection would require investigators to adopt an unfamiliar and unpromising approach - to search deliberately for supposedly inferior materials (incomplete and eroded footprints) in the hope of finding information which is generally believed to be unimportant or potentially misleading (adventitious features). In short, it may be the case that certain features of substrate deformation have gone unnoticed because there is no incentive to search for them.

**Figure 30:**
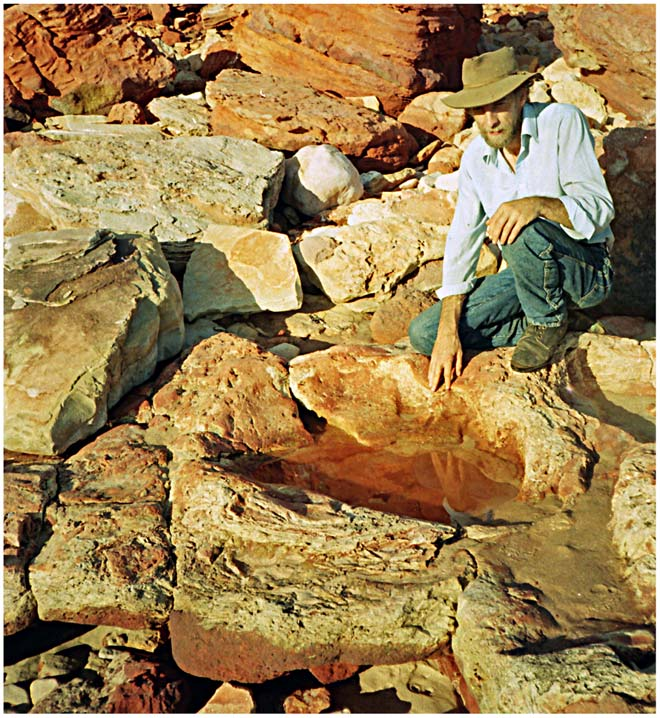
Sauropod pes print, cf. ichnogenus *Brontopodus*, in silicified carpet of plant debris overlying red palaeosol. This non-layered substrate does not register any transmitted reliefs. Note conspicuous traces of claws along the lateral edge of the print.

Second, features of transmitted relief are more easily detected in some settings than in others, and in that respect the thinly and evenly-bedded lagoonal substrates of the Broome Sandstone are practically ideal. By contrast more thickly-bedded and homogeneous substrates, such as those of the Glen Rose Formation of Texas, are not so well-suited to recording and displaying patterns of sub-surface deformation (e.g. [43], figure 34.10]). The significance of this lithological control is apparent elsewhere in the Broome Sandstone, where sauropods left their tracks in mottled palaeosols and carpets of silicified plant debris (Figure 30). Those non-layered substrates do not register and exhibit any transmitted reliefs, even though the sauropod tracks impressed into them are just as large and as well-preserved as those in lagoonal substrates nearby. Even so, sauropod tracks at some sites around the world are preserved in substrates that are potentially suitable for the development of transmitted reliefs. For instance, sauropod tracks in thinly-bedded sandstones of Early Cretaceous (Berriasian) age at Münchehagen, in Germany, do show some clear indications of transmitted relief. In fact, Lockley et al. ([34], figure 4) illustrated one manus-pes couple which appears to be enclosed in a very shallow basin shaped like a figure 8, rather like some of the larger saddle-shaped basins illustrated here (e.g. Figure 20). Such a clue suggests that there might be even closer parallels to the patterns of deformation seen in the Broome Sandstone, though these are unlikely to be detected by the conventional search for pristine footprints on an intact bedding plane.

The sheer abundance of footprints in the Broome Sandstone indicates that sauropods were common visitors to the lagoonal environments bordering the coastal plain of north-western Australia at the start of the Cretaceous. As the lagoons were devoid of vegetation (except, perhaps, for algae), they were clearly not sources of food, and it seems more likely that they were exploited as a convenient route by sauropods travelling along the coastal plain from one feeding-ground to the next. Presumably those extensive lagoons afforded relatively safe and easy transit, as there would have been no concealment for predators and no steep slopes or other obstacles to be negotiated. Although the track-makers’ feet sometimes sank very deeply into the unconsolidated lagoonal sediments, there are very few examples of messy sauropod tracks, and no evidence at all that any of the animals ever became mired.

At some spots the sauropod tracks are aligned in parallel and so densely packed as to be suggestive of animals moving in groups. Moreover it seems quite evident that the sauropod track-makers adhered to well-trodden routes or thoroughfares (Figure 26) while avoiding the intervening areas (e.g. Figures 23,25A). That tendency to follow a well-trodden path might reflect nothing more than an obligation imposed by their immense size and weight. Animals as ponderous as sauropods would probably have been reluctant to traverse wet and potentially slippery slopes, as are elephants today when they approach river banks and the margins of water-holes. If sauropods were as wary as elephants in negotiating sloping terrain, they would naturally have tended to walk on the lower and safer ground - which, in practice, would be any area that was already trodden by earlier visitors. In doing so, they would automatically have followed, deepened and widened the routes pioneered by their predecessors, thereby reshaping the topography of the landscape they inhabited.
